# Redescription and molecular characterisation of the fish ectoparasite *Anilocra capensis* Leach, 1818 (Isopoda: Cymothoidae), with description of six new species of *Anilocra* Leach, 1818 from Africa

**DOI:** 10.1186/s13071-019-3578-5

**Published:** 2019-08-01

**Authors:** Rachel L. Welicky, Nico J. Smit

**Affiliations:** 1 Water Research Group, Unit for Environmental Sciences and Management, Potchefstroom Campus, North-West University, Private Bag X6001, Potchefstroom, 2520 South Africa; 2 Present Address: School of Aquatic and Fishery Sciences, University of Washington, 1122 NE Boat Street, Seattle, WA 98105 USA

**Keywords:** Africa, *Anilocra*, Cymothoid, Fish parasite, Molecular characterization, Morphological description

## Abstract

**Background:**

*Anilocra capensis* Leach, 1818 is the only named species of *Anilocra* Leach, 1818 from South Africa. *Anilocra* is a large genus (> 40 species) with high levels of diversity reported from the Caribbean and Indo-West Pacific. Considering it is highly unlikely that all records of *Anilocra* from South Africa can be of a single species, the aim of this study was to better understand the diversity of *Anilocra* from this region and continent.

**Methods:**

To redescribe *A. capensis*, the syntypes of *A. capensis* and specimens recorded as *A. capensis* from Africa were borrowed from the Natural History Museum, London, UK, and The iZiko South African Museum, Cape Town. Newly collected fresh samples of *A. capensis* were collected from off Cape Town, South Africa. Morphological redescriptions of the syntypes, and other museum and fresh material were conducted. Fresh samples were used to characterise molecularly *A. capensis* using the mitochondrial cytochrome *c* oxidase subunit 1 gene (*cox*1).

**Results:**

Morphological analyses demonstrated that apart from *A. capensis* there are six *Anilocra* species new to science from Africa: *Anilocra ianhudsoni* n. sp., *Anilocra bunkleywilliamsae* n. sp., *Anilocra paulsikkeli* n. sp., *Anilocra jovanasi* n. sp., *Anilocra angeladaviesae* n. sp. and *Anilocra hadfieldae* n. sp. Of the species under study, specimens of *A. capensis* appear to demonstrate the most individual variation, which occurs in pleonite width, pleotelson form and uropod length. We determined that African species of *Anilocra* can be primarily differentiated by the proportional shape and size of the full body in dorsal view and pereonites 1, 6 and 7. Other defining morphological traits include proportional shape and size of the pereopods, and the antenna and antennula peduncles. Lastly, the molecular characterisation of *A. capensis* is provided and the interspecific divergence with Mediterranean species is smaller than that with Caribbean species.

**Conclusions:**

The results of this study provide a detailed redescription of *A. capensis* and the first molecular barcode for this organism. Six new species of *Anilocra* from Africa are described, establishing that the diversity of *Anilocra* in this region is greater than previously known. With this new understanding of species differences, we can accurately conduct detailed molecular and ecological analyses of *Anilocra* from Africa with certainty of the organism under study.

## Background

Members of the isopod fish parasitic family Cymothoidae Leach, 1818 conspicuously infest the skin, buccal cavity, or gill chamber of their hosts. This family has a global distribution and there are more than 43 recognised genera [1]. One of the most commonly observed genera is *Anilocra* Leach, 1818, and it remains one of the best studied ecologically [2–4]. In fact, *Anilocra* was the first genus of a marine fish parasite to be described from South Africa [5].

Leach [6] erected *Anilocra* to accommodate three species. One of these species was *Anilocra capensis* Leach, 1818, collected in the Cape region of South Africa. In Schioedte & Meinert’s (1879) redescription of *A. capensis*, the authors provided only two dorsal view drawings of the female, and one dorsal view drawing of the male [7]. Schioedte & Meinert (1879) did not examine Leach’s type-material. Since 1879, no further supplemental redescription of *A. capensis* has been published. As a result, nearly all reports of *Anilocra* infestation on South African marine fishes have been recorded as *A. capensis.*

The majority of *Anilocra* have been reported from tropical environments, e.g. [8–10]. Although cymothoids are generally more speciose in tropical than in temperate environments, temperate systems are nonetheless diverse. Accordingly, *Anilocra* diversity in South Africa should be far greater than the single recorded species. This should particularly be the case as South Africa’s coastline encompasses two oceans that span temperate to subtropical ecosystems. Moreover, these ecosystems are suitable for a diversity of potential fish hosts.

To test the aforementioned prediction, a review of *A. capensis* based on the syntypes and all other available material was warranted. We seek to provide descriptions of species of *Anilocra* from South Africa and the African continent in order to clarify and describe the diversity of *Anilocra* from the eastern Atlantic and western Indian Oceans. Moreover, we aim to provide molecular data for *A. capensis* so that future taxonomic studies can at a minimum describe *Anilocra* from Africa as *A. capensis* or a different species of *Anilocra.*

## Methods

### Specimen collection

In March 2017, specimens identified as *Anilocra capensis* with locality data from Africa, along with the syntypes of *A. capensis* were borrowed from the Natural History Museum, London, UK (NHM). We examined other specimens of *Anilocra* from outside South Africa from NHM to confirm or reject if they were *A. capensis*. In July 2017, all specimens labelled as *Anilocra* from The iZiko South African Museum, Cape Town (SAM) were borrowed. In April 2018, live *A. capensis* were collected from the Hottentot seabream, *Pachymetopon blochii* Valenciennes, at two localities: Cape Town Harbour wall and the False Bay Yacht Club harbour. Infested fishes were speared on SCUBA under the direction and permitting of Two Oceans Aquarium. The fresh material was preserved in 80% ethanol for later morphological analysis (see Table 1 for sample sizes and locality data). For some specimens, leg tissue was extracted using forceps and placed in 96% ethanol for molecular analysis.

**Table 1:** Details of *Anilocra* species examined

	Collection locality	Male	Female
*Anilocra capensis*	Off Cape Town (Harbor Wall), South Africa	8	9
	False Bay, Cape Town, South Africa	26	38
	Table Bay, Cape Town, South Africa		7
	Cape Town region, South Africa		5
*Anilocra ianhudsoni* n. sp.	Nahoon River, East London, South Africa	1	2
*Anilocra bunkleywilliamsae* n. sp.	Mawalana Estuary, South Africa	1	2
*Anilocra paulsikkeli* n. sp.	Delagoa Bay, Mozambique	2^a^	2
*Anilocra jovanasi* n. sp.	Delagoa Bay, Mozambique		1
*Anilocra angeladaviesae* n. sp.	Off Cape Blanc, Morocco	2	4
*Anilocra hadfieldae* n. sp.	Off Cape Blanco, Gambia	1	1

^a^One specimen may be transitional

### Molecular analysis

Genomic DNA was extracted from 8 of the freshly collected *A. capensis*. A rapid DNA extraction method as described in the KAPA Express Extract Kit (Kapa Biosystems, Cape Town, South Africa) was used. Polymerase chain reactions (PCR) were used to amplify a 710 bp fragment of the mitochondrial cytochrome *c* oxidase subunit gene (*cox*1) using the primer sets LCO 1490 and HCO 2198 [11]. PCR was performed using 12.5 μl Thermo Scientific DreamTaq PCR master mix (2×) (2× DreamTaq buffer, 0.4 mM of each dNTP, and 4 mM MgCl_2_), 1.25 μl of each primer, 1 μl DNA, and 9 μl of PCR-grade nuclease free water (Thermo Fisher Scientific, Vilnius, Lithuania). Total volume per reaction was 25 μl, and PCR reactions were conducted using a ProFlex™ PCR thermal cycler (Applied Biosystems by Life Technologies). Reactions were amplified under the following PCR conditions: Stage 1, 94 °C for 5 min, Stage 2, 36 cycles of 94 °C for 30 s, 47 °C for 50 s, 72 °C for 2 min, and Stage 3, 72 °C for 10 min. PCR products were sent to a commercial sequencing company (Inqaba Biotechnical Industries (Pty) Ltd, Pretoria, South Africa) for purification and sequencing in both directions. Obtained sequences were assembled, and chromatogram-based contigs were generated using Geneious Ver. 9.1. Sequences were aligned and trimmed to the length of the shortest sequence using MEGA 7 software program [12].

Using BLASTn (Basic Local Alignment Search Tool; http://www.ncbi.nlm. nih.gov/blast), the obtained sequences were verified as belonging to the Isopoda. Mean intraspecific divergence for *A. capensis* and interspecific divergence with all other *Anilocra* reported on GenBank were examined using p-distance and number of bp differences. We utilised the following sequences: *Anilocra physodes* Linneaus, 1758 (EF455817); *Anilocra chromis* Williams & Williams, 1981 (KY5622739); *Anilocra brillae* Welicky, Hadfield, Sikkel & Smit, 2017 (KY562744); *Anilocra haemuli* Williams & Williams, 1981 (KY562752); *Anilocra prionuri* Williams & Bunkley-Williams, 1986 (LC15954); and *Anilocra clupei* Williams & Bunkley-Williams, 1986 (LC160309). Newly-generated sequences for *Anilocra capensis* were deposited in the GenBank database under the accession numbers MK450443-MK450452.

### Morphological data

Specimens of *Anilocra* were processed according to the techniques described in [13, 14]. Dissection was permitted for specimens from SAM but not the NHM. Regarding the museum collection specimens in this study, SAM assigns one accession number per species and collection location, whereas NHM assigned one accession number per individual in most cases. Accession numbers from NHM are indicated with the abbreviation BMNH. Descriptions were prepared using DELTA (Descriptive Language for Taxonomy) [15] using a general character set for the Cymothoidae [16, 17]. Ratios and measurements were rounded off to one decimal place and were made using maximum values of the specific measured article. Measurements were taken and ratios determined from ovigerous and non-ovigerous female (♀), and male (♂) specimens. Pleotelson length (TL) and width (W) for all specimens examined are reported as TL × W. All measurements are reported in millimeters (mm). Higher order classification follows Brandt & Poore [18]. Fish nomenclature follows FishBase [19].

To comply with the regulations set out in article 8.5 of the amended 2012 version of the *International Code of Zoological Nomenclature* (ICZN [20], details of all new taxa have been submitted to ZooBank. For each new taxon, the Life Science Identifier (LSID) is reported in the taxonomic summary.

## Results

### Specimen summary

We identified from the museum loans and field collection *A. capensis* and six new species of *Anilocra*. These specimens can be distinguished using the key provided here. Sample sizes and locality data for each species are given in Table 1.

### Molecular analyses

Comparative sequence analysis indicated that the intraspecific divergence of *A. capensis* was 1–7 nt (0.04%). The interspecific divergence between *A. capensis* and *A. physodes* was 63–74 nt (15.5%). The interspecific divergence between *A. capensis* and Caribbean species of *Anilocra* were: *A. chromis*: 138–157 nt (24.9%), *A. brillae*: 138–157 nt (24.9%), *A. haemuli*: (KY562752) 136–154 (24.4%), *A. prionuri*: (LC159541) 136–157 (24.8%), and *A. clupei*: (LC160309) 132–144 (24.4%).

### Taxonomy

**Genus** ***Anilocra*** **Leach, 1818**

***Diagnosis.*** Diagnosis and synonymy in Bruce [21]. Body dorsally symmetric, weakly to strongly vaulted. Cephalon non-tri-lobed to weakly tri-lobed, anterior of frontal margin of rostrum blunt and folded down posteriorly between antennule bases. Antennula shorter than antenna. Pereonite 5 or 6 widest, coxae 4–6 longer, more anteriorly pointed than 1–3. Pereopods 1–7 increase in size. *Type-species*: *Anilocra cuvieri* Leach, 1818.

**Remarks**

Leach [6] described three species: *Anilocra cuvieri* Leach, 1818, *Anilocra mediterranea* Leach, 1818 and *Anilocra capensis. Anilocra cuvieri* was designated as the type-species by Kussakin [22]. There are three *Anilocra capensis* syntypic specimens held at The Natural History Museum, London, presented by Leach from the Cape of Good Hope (see ‘Material examined’) [23].

Nearly half of the species of *Anilocra* accepted today were described in the 1980s, with many originating from the Indo-Pacific and Caribbean [8, 21]. Since 2001, just four new species have been described based on material from the Caribbean [24], Chile [25], Lebanon [26] and the Gulf of Mexico [27]. In 2017, using morphological and molecular data, *Anilocra brillae* [24] from the Caribbean was identified and described based on specimens that were originally thought to be *Anilocra haemuli* [8]. This revision is an example of the cryptic nature of some *Anilocra* spp., and the importance of combining morphological and molecular data whenever possible.

Of the skin-attaching cymothoids, species of *Anilocra* most closely resemble species of *Renocila* Miers, 1880 and *Nerocila* Leach, 1818, but generic level differences can be distinguished without dissection or stereoscopic microscope [28–30]. Briefly, in *Anilocra* spp. pereopod 7 is longer than 6, but in *Renocila* spp. pereopod 6 is similar in length to 7. The rostrum is not ventrally folded between the antennae in *Renocila* spp., but it is in *Anilocra* spp. The cephalon of *Nerocila* spp. is posteriorly strongly trilobed, whereas the cephalon of *Anilocra* spp. is not tri-lobed to very weakly tri-lobed. Further comparisons among cymothoid genera and generic features are detailed in Welicky et al. [24].

***Anilocra capensis*** **Leach, 1818**

***Type-host***: Probable host *Pachymetopon blochii* referred to as “Sargus Hottentotus” in Schiöedte & Meinert (1883) [7].

***Type-material***: BMNH 1979.329:207b (♀ 43.0 × 18.0) (designated as the lectotype in the present study); BMNH 1979.329:207c (♀ 46.0 × 19.5); BMNH 1979.329:207a (♂ 35.0 × 10.0). Paralectotypes: BMNH 1979.329:207c (♀ 46.0 × 19.5), BMNH 1979.329:207a (♂ 35.0 × 10.0). Presented by W. Leach in 1818 from Cape of Good Hope.

***New material examined***: From Cape Town Harbor Wall (33°53′58″S, 18°25′35″E) SAMC-A091297: ♂ (22.0 × 7.0; 25.0 × 7.0; 26.0 × 8.5; 27.0 × 8.0; 27.0 × 8.5; 29.0 × 9.0; 31.5 × 9.0; 33.0 × 10.0), non-ovigerous ♀ (43.5 × 16.5; 44.5 × 20.0), ovigerous ♀ (43.0 × 18.5; 44.0 × 19.5; 45.0 × 19.5; 45.0 × 19.5; 49.0 × 20.0; 49.0 × 20.0), damaged ♀ (46.6 × 19.5). From False Bay Yacht Club Marina (34°11′32″S, 18°26′01″E) SAMC-A091298: ♂ (12.0 × 3.5; 15.0 × 4.0; 18.0 × 5.0; 18.5 × 5.0; 19.0 × 4.5; 19.0 × 5.0; 19.5 × 5.5; 20.0 × 5.0; 21.0 × 5.5; 21.0 × 5.5; 21.0 × 6.0; 22.0 × 6.0; 23.0 × 6.0; 23.0 × 6.5; 23.0 × 7.0; 23.0 × 8.0; 23.6 × 6.5; 24.0 × 5.0; 24.0 × 6.5; 24.0 × 7.0; 24.0 × 7.0; 25.0 × 7.0; 28.0 × 8.0; 28.0 × 8.0; 28.5 × 8.0; 29.0 × 9.0), non-ovigerous ♀ (26.0 × 10.0; 27.0 × 9.5; 27.5 × 9.5; 29.5 × 9.0; 29.5 × 9.0; 34.0 × 13.0; 35.5 × 14.0; 39.0 × 13.0; 42 × 18; 43.5 × 16.5; 44.5 × 20.5; 51.5 × 23.0), ovigerous ♀ (28.0 × 12.0; 29.0 × 11.0; 34.5 × 13.0; 34.5 × 15.0; 36.0 × 16.0; 37.5 × 15.0; 38.0 × 15.0; 38.0 × 15.5; 38.0 × 15.5; 39.0 × 14.0; 40.0 × 15.0; 40.0 × 15.0; 41.0 × 15.0; 43.0 × 18.5; 43.5 × 17.0; 47.0 × 19.0; 50.5 × 25.0), damaged ♀ (27.0 × 9.5; 29.5 × 12.0; 32.0 × 13; 37.0 × 14.0; 37.0 × 14.0; 37.0 × 17.0; 37.5 × 14.0; 40.0 × 18.0). All specimens collected by R. L. Welicky, O. Kudlai, A. Greyling, D. van Rooyen, T. Beukes and Two Oceans Aquarium personnel on 28–29 April 2017 on the host *P. blochii*.

***Additional museum material examined***: Collected by UCT Ecological Survey from Table Bay on host *P. blochii* and held at SAM as SAMA44882: ♀ (44.0 × 18.0; 44.0 × 20.0; 47.0 × 19.0; 47.0 × 21.0; 49.0 × 21.0; 50.0 × 22.0; 53.0 × 22.0). Collected from SAM and not catalogued: ♀ (45.0 × 22.0; 49.0 × 19.0; 49.0 × 20.0; 50.0 × 20.0; 50.0 × 22.0).

***Representative DNA sequences***: MK450443-MK450452

**Description**

**Fig. 1:**
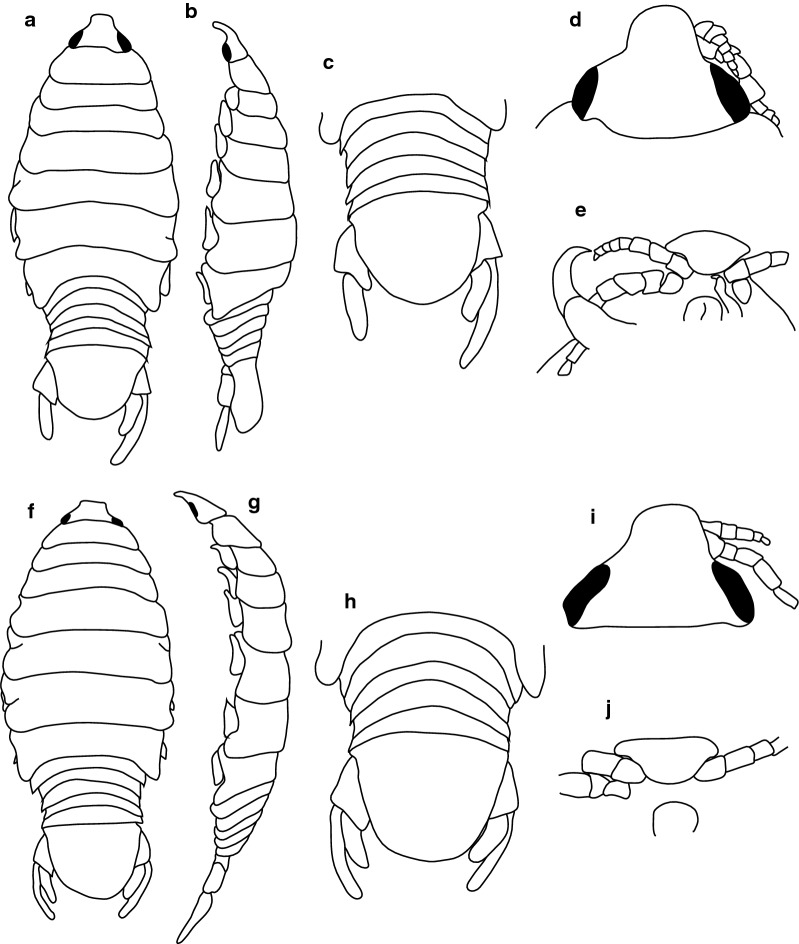
*Anilocra capensis* ♀ (**a**–**e** 43.0 × 18.0, BMNH 1979.329:207b; **f**–**j** 46.0 × 19.5, BMNH 1979.329:207c). **a** Dorsal view. **b** Lateral view. **c** Pleotelson. **d** Dorsal view of cephalon. **e** Ventral view of cephalon. **f** Dorsal view. **g** Lateral view. **h** Pleotelson. **i** Dorsal view of cephalon. **j** Ventral view of cephalon

***Female lectotype.*** [BMNH 1979.329:207b; Fig. 1.] Size 43.0 × 18.0. Body ovoid, 2.6 times as long as greatest width; dorsal surfaces smooth and polished in appearance; widest at pereonite 5, most narrow at pereonite 1; pereonite lateral margins mostly posteriorly ovate. Cephalon 0.6 as long as wide as measured in dorsal view, trapezoid-shaped. Pereonite 1 smooth, anterior border straight, anterolateral angle narrowly rounded, in line with base of cephalon. Posterior margins of pereonites smooth, slightly curved laterally. Coxae 2–3 wide, with posteroventral angles rounded; coxae 4–7 with rounded point, curved, not extending past posterior pereonite margin; coxa 7 extending past anterior pereonite margin, coxae 4–6 not extending past anterior pereonite margin. Pereonites 1–5 increasing in length and width; pereonites 6–7 decreasing in length and width; pereonites 5 and 6 subequal, or pereonites 1–4 narrower. Pleon with pleonite 5 nearly widest, visible in dorsal view; posterior margin of pleonites 1–4 posteriorly concave, pleonite 5 posteriorly slightly concave to straight or smooth, mostly concave. Pleonite 2 not laterally overlapped by pereonite 7; posterolateral angles of pleonite 2 narrowly rounded. Pleonite 1 similar in form to pleonite 2 and 3. Pleonites 3–5 similar in form to pleonite 2; pleonite 5 widest or nearly widest to pleonite 4, not overlapped by lateral margins of pleonite 4 or with posterolateral angles narrowly rounded, posterior margin straight. Pleotelson 0.8 times as long as anterior width, dorsal surface smooth; lateral margins convex, posterior margin evenly rounded.

Antennula approximately as stout as antenna, comprised of 8 articles; peduncle articles 1 and 2 distinct, articulated. Antenna comprised of 8 articles.

Uropod more than half the length of pleotelson, peduncle 0.4 times as long as rami, peduncle lateral margin without setae or without medial short acute robust seta; rami extending beyond pleotelson, marginal setae absent, apices broadly rounded. Endopod apically rounded, 3.6 times as long as greatest width, lateral margin weakly convex, medial margin weakly convex. Exopod extending beyond end of endopod, 7.2 times as long as greatest width, apically rounded, lateral margin weakly convex, medial margin weakly convex, terminating without setae.

***Female paralectotype.*** [BMNH 1979.329:207c; Fig. 1.] Size 46.0 × 19.0. Body ovoid, 2.6 times as long as greatest width; dorsal surfaces smooth and polished in appearance; widest at pereonite 5, most narrow at pereonite 1; pereonite lateral margins mostly posteriorly ovate. Cephalon 0.32 as long as wide as measured in dorsal view, trapezoid-shaped. Eyes oval, with distinct margins. Pereonite 1 smooth, anterior border straight, anterolateral angle narrowly rounded, in line with base of cephalon. Posterior margins of pereonites smooth and slightly curved laterally. Coxae 2–3 wide; with posteroventral angles rounded; coxae 4–7 with rounded point and curved; not extending past posterior pereonite margin, coxa 7 extending past anterior pereonite margin, coxae 4–6 not extending past anterior pereonite margin. Pereonites 1–5 increasing in length and width; pereonites 6–7 decreasing in length and width; pereonites 5 and 6 subequal, pereonites 1–4 narrower. Pleon with pleonite 1 wider than pleonites 2–5, visible in dorsal view; posterior margin of pleonites 1–4 posteriorly concave; pleonite 5 posteriorly slightly concave to straight or smooth, mostly concave. Pleonite 2 not overlapped by pereonite 7; posterolateral angles of pleonite 2 narrowly rounded. Pleonite 1 similar in form to pleonites 2 and 3. Pleonites 3–5 similar in form to pleonite 2; pleonite 5 equal in width to pleonite 4 or free, not overlapped by lateral margins of pleonite 4, pleonite 5 with posterolateral angles narrowly rounded, posterior margin straight. Pleotelson 0.9 times as long as anterior width, dorsal surface smooth, lateral margins convex, posterior margin evenly rounded.

Antennula approximately as stout as antenna; peduncle articles 1 and 2 distinct and articulated.

Uropod more than half the length of pleotelson, peduncle 0.7 times as long as rami, peduncle lateral margin without setae; rami extending beyond pleotelson, marginal setae absent, apices broadly rounded. Endopod apically rounded, 3.8 times as long as greatest width, lateral margin weakly convex, medial margin weakly convex. Exopod extending beyond end of endopod, 7 times as long as greatest width, apically rounded, lateral margin weakly convex, medial margin weakly convex, terminating without setae.

**Fig. 2:**
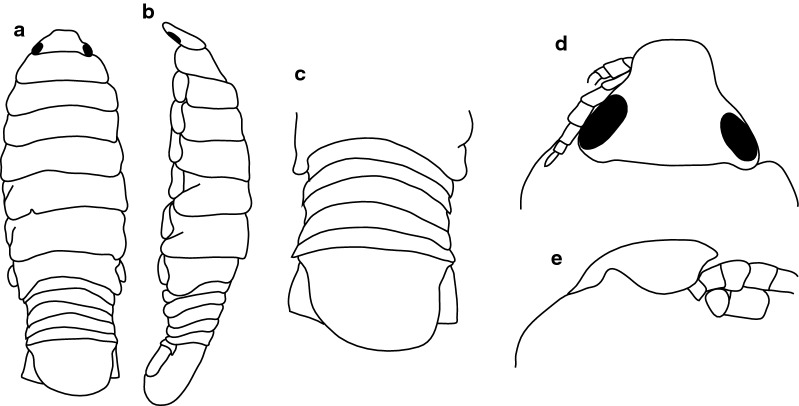
*Anilocra capensis* ♂ (35.0 × 10.0, BMNH 1979.329:207a). **a** Dorsal view. **b** Lateral view. **c** Pleotelson. **d** Dorsal view of cephalon. **e** Ventral view of cephalon

***Male paralectotype.*** [BMNH 1979.329:207a; Fig. 2.] Size 35.0 × 10.0. Body similar to female but smaller and narrower; 3.5 times as long as wide. Pleopod 2 *appendix masculina* distally narrowly rounded.

**Fig. 3:**
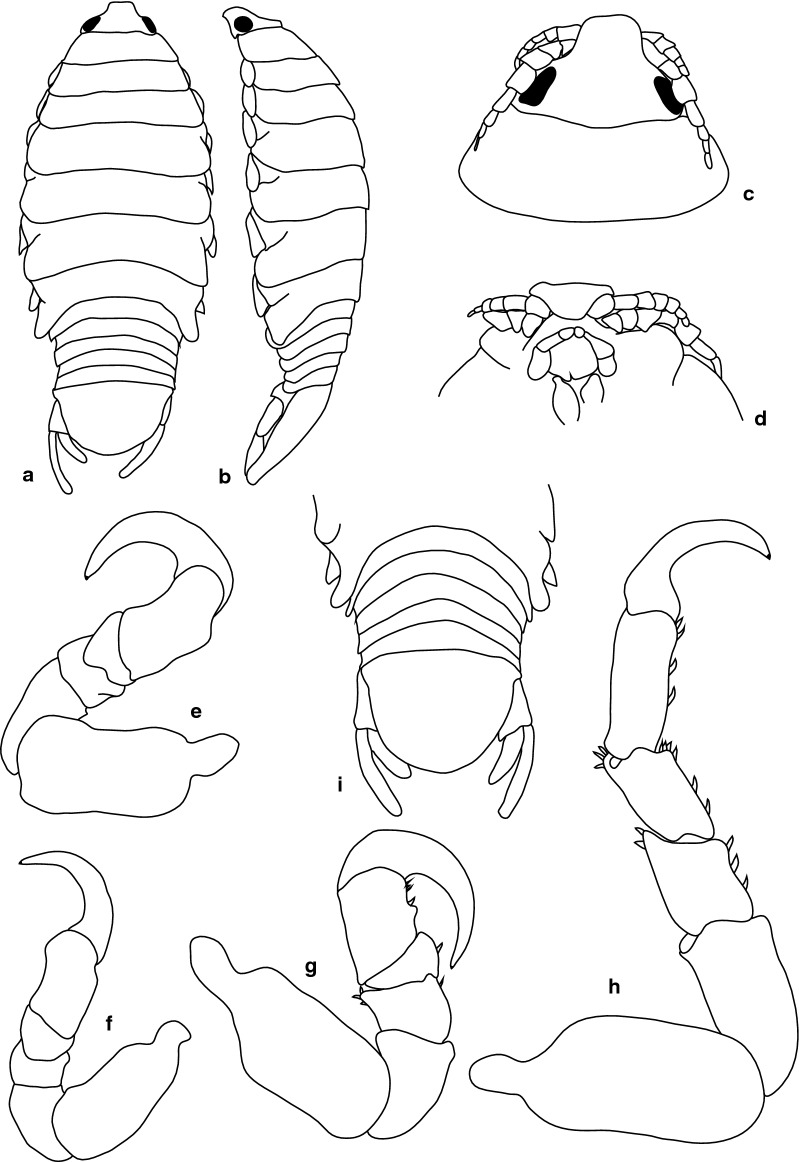
*Anilocra capensis* ♀ (50.0 × 22.0, SAMC-A091297). **a** Dorsal view. **b** Lateral view. **c** Dorsal view of cephalon. **d** Ventral view of cephalon. **e** Pereopod 1. **f** Pereopod 2. **g** Pereopods 3. **h** Pereopod 6. **i** Pleotelson

**Fig. 4:**
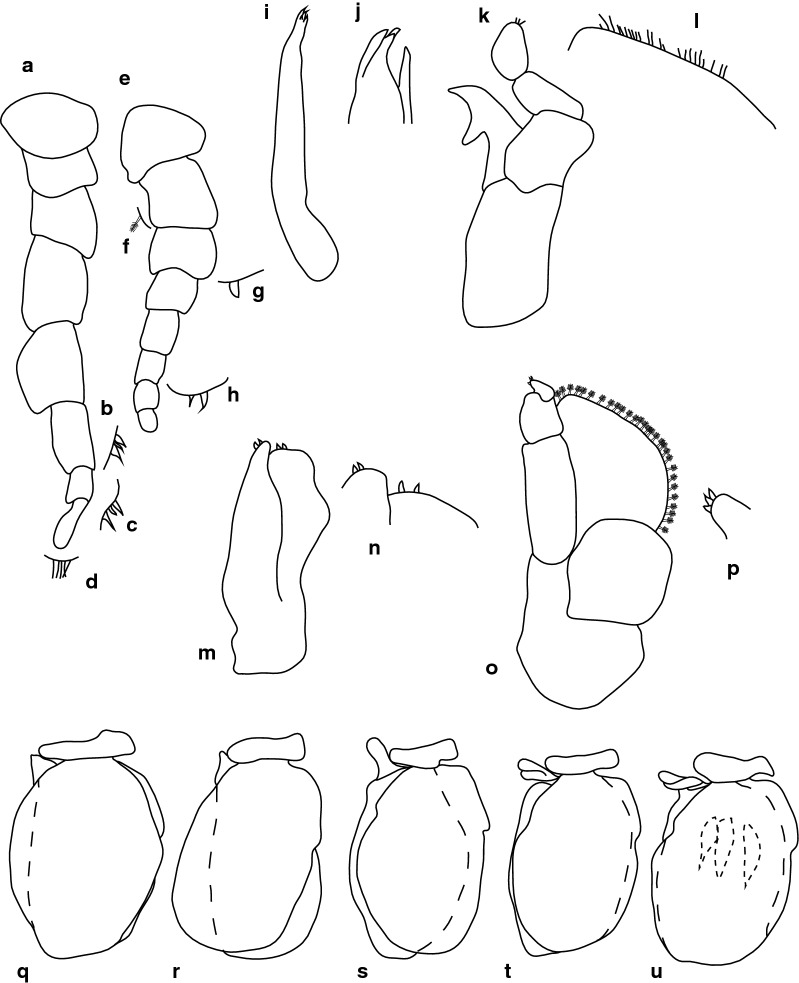
*Anilocra capensis* ♀ (45.0 × 22.0, SAMC-A091297). **a**–**d** Antenna. **a** Antenna. **b** Article 6 robust setae. **c** Article 7 robust setae. **d** Article 8 simple setae. **e**–**h** Antennula. **e** Antennula. **f** Plumose setae article 2. **g** Robust setae article 3. **h** Robust setae article 6. **i** Maxillule. **j** Maxillule apex. **k** Mandible. **l** Article 3 mandibular palp. **m** Maxilla. **n** Maxilla apex. **o** Maxilliped. **p** Article 3 of maxilliped. **q**–**u** Pleopods 1–5, respectively

***Female***. [New material SAMC-A091297; Figs. 3, 4.] Size 45.0 × 22.0. Body ovoid, 2.4 times as long as greatest width; dorsal surfaces smooth and polished in appearance; widest at pereonite 5, most narrow at pereonite 1; pereonite lateral margins mostly posteriorly ovate. Cephalon 0.42 as long as wide as measured in dorsal view, trapezoid-shaped. Eyes oval, with distinct margins, one eye width 0.2 times width of cephalon, one eye length 0.4 times length of cephalon. Pereonite 1 smooth, anterior border straight, anterolateral angle narrowly rounded, in line with base of cephalon. Posterior margins of pereonites smooth, slightly curved laterally. Coxae 2–3 wide, with posteroventral angles rounded; coxae 4–7 with rounded point and curved; coxae 4–6 not extending past posterior pereonite margin; coxa 7 extending past anterior pereonite margin; coxae 4–6 not extending past anterior pereonite margin. Pereonites 1–5 increasing in length and width; pereonites 6–7 decreasing in length and width; pereonites 5 and 6 subequal, pereonites 1–4 narrower. Pleon with pleonite 1 wider than pleonites 2–5, visible in dorsal view; posterior margin of pleonites 1–4 posteriorly concave, pleonite 5 posteriorly slightly concave to straight or smooth, mostly concave. Pleonite 2 not overlapped by pereonite 7; posterolateral angles of pleonite 2 narrowly rounded. Pleonite 1 similar in form to pleonites 2 and 3. Pleonites 3–5 similar in form to pleonite 2; pleonite 5 equal width to pleonite 4 or free, not overlapped by lateral margins of pleonite 4, with posterolateral angles narrowly rounded, posterior margin straight. Pleotelson 0.8 times as long as anterior width, dorsal surface smooth, lateral margins convex, posterior margin evenly rounded.

Antennula approximately as stout as antenna, comprised of 8 articles; peduncle articles 1 and 2 distinct, articulated; article 2 0.8 times as long as article 1; article 3 0.7 times as long as wide, 0.4 times as long as combined lengths of articles 1 and 2; antennula flagellum with 5 articles, extending to middle of eye; peduncle article 3 0.8 times as long as wide, article 2 with plumose setae, or articles 3 and 6 with robust setae. Antenna comprised of 8 articles, peduncle article 3 1.4 times as long as article 2, article 4 1.3 times as long as wide, article 5 1.5 times as long as wide, 1.0 times as long as article 4; antenna flagellum with 3 articles, terminal article terminating in 1–5 short simple setae, extending to middle of pereonite 1. Mandibular molar process ending in an acute incisor, with 23 simple setae. Maxillula simple, with 4 terminal robust setae. Maxilla medial lobe partly fused to lateral lobe; 2 recurved robust setae; and 2 large recurved robust setae. Maxilliped weakly segmented, with lamellar oostegite lobe or second, smaller oostegite lobe on basal part of article, article 3 with 3 recurved robust setae.

Pereopod 1 basis 0.4 times as long as greatest width; ischium 1.9 times as long as basis; merus proximal margin without bulbous protrusion; carpus with straight proximal margin; propodus 2.0 times as long as wide; dactylus stout, 2.4 times as long as basal width. Pereopod 2 propodus 1.8 as long as wide; dactylus 1.3 as long as propodus. Pereopods gradually increasing in size towards posterior. Pereopod 6 basis 2.6 times as long as greatest width, ischium 0.4 times as long as basis, propodus 1.3 as long as wide, dactylus 1.4 as long as propodus. Pereopod 7 basis 2.6 times as long as greatest width; ischium 0.6 as long as basis, without protrusions; merus 1.1 times as long as wide, 0.5 as long as ischium; carpus 1.3 times as long as wide, 1 as long as ischium, without bulbous protrusion; propodus 2.4 times as long as wide, 0.7 as long as ischium; dactylus moderately slender, 1.1 times as long as propodus, 2.9 times as long as basal width.

Pleopods without setae or lobes, exopod larger than endopod. Pleopod 1 exopod 1.3 times as long as wide, lateral margin weakly convex, distally narrowly rounded, medial margin weakly oblique, medial margin weakly convex; endopod 1.2 times as long as wide, lateral margin weakly convex, distally narrowly rounded, medial margin slightly convex, peduncle 4.5 times as wide as long, without retinaculae. Pleopods 2–5 similar to pleopod 1. Pleopods 3–5 endopods proximal borders do not extend below exopod to peduncle. Peduncle lobes absent on pleopods 1–3, or present, increasing on pleopods 4–5.

Uropod more than half the length of pleotelson, peduncle 0.5 times as long as rami, peduncle lateral margin without setae or without medial short acute robust seta; rami extending beyond pleotelson, marginal setae absent, apices broadly rounded. Endopod apically rounded, 3.5 times as long as greatest width, lateral margin weakly convex, medial margin weakly convex. Exopod extending beyond end of endopod, 6 times as long as greatest width, apically rounded, lateral margin weakly convex, medial margin weakly convex, terminating without setae.

**Fig. 5:**
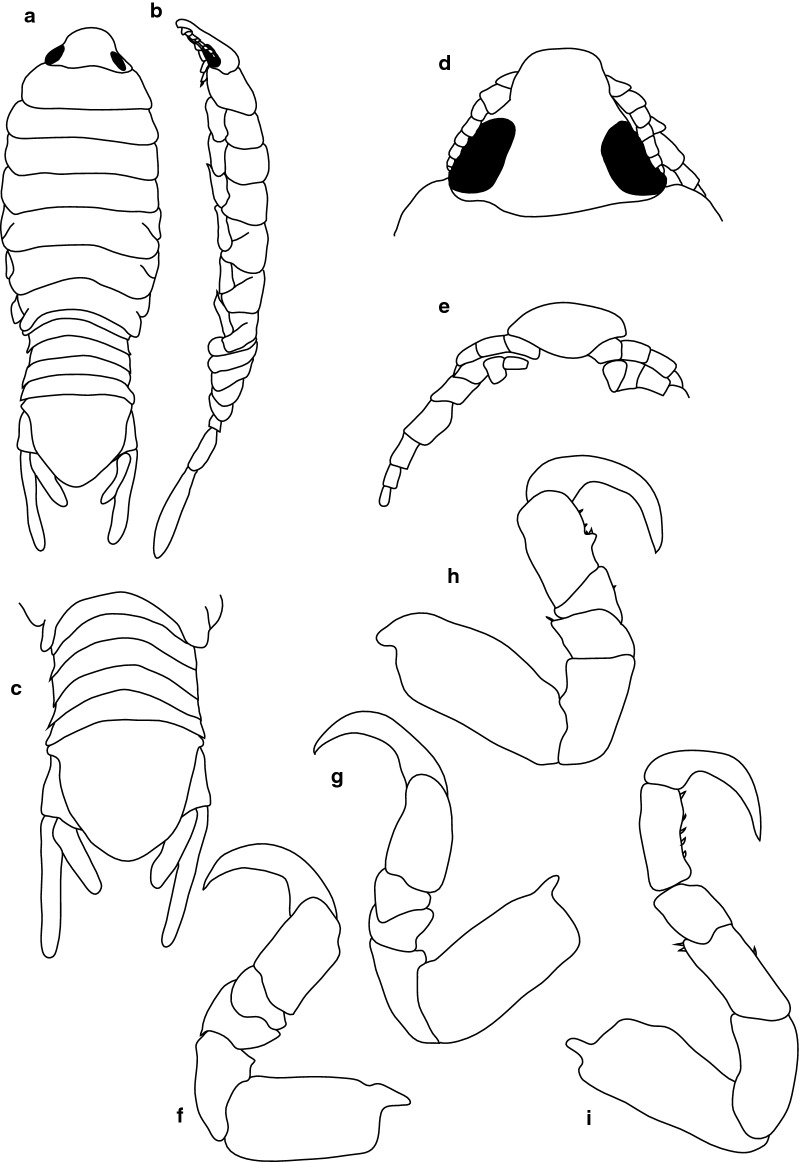
*Anilocra capensis* ♂ (28.0 × 8.0, SAMC-A091297). **a** Dorsal view. **b** Lateral view. **c** Pleotelson. **d** Dorsal view of cephalon. **e** Ventral view of cephalon. **f** Pereopod 1. **g** Pereopod 2. **h** Pereopod 6. **i** Pereopod 7

**Fig. 6:**
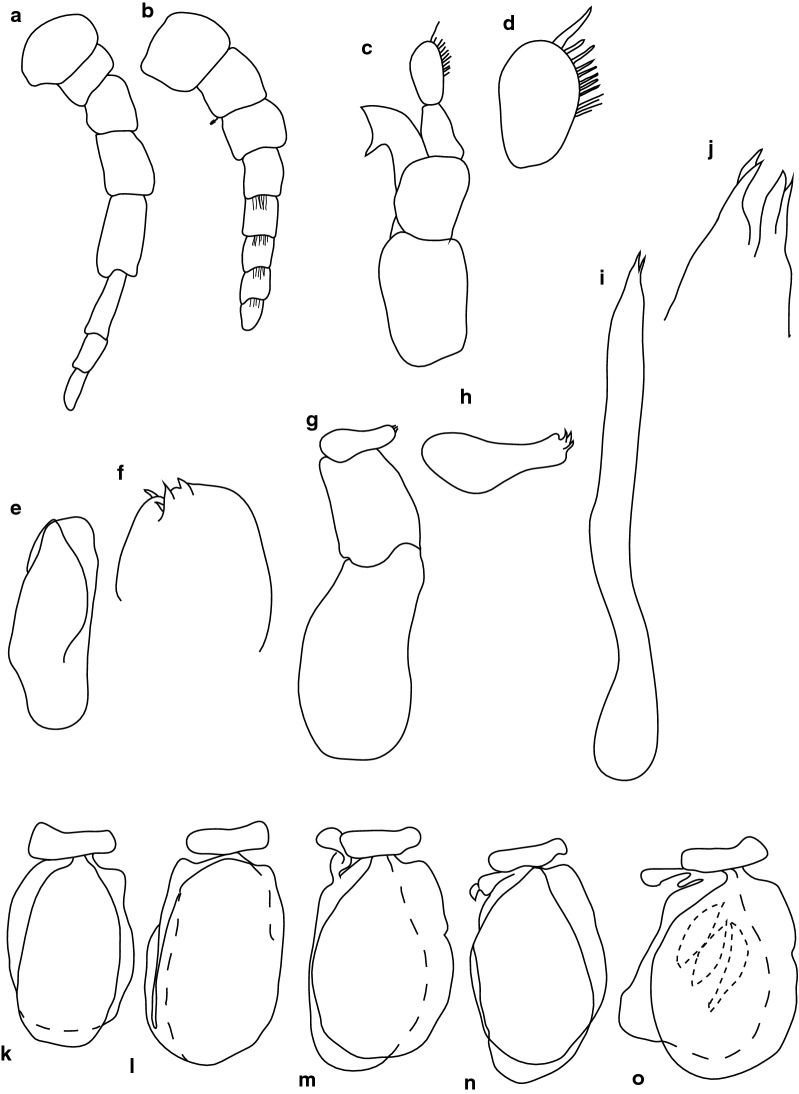
*Anilocra capensis* ♂ (28.0 × 8.0, SAMC-A091297). **a** Antenna. **b** Antennula. **c** Mandible. **d** Article 3 mandibular palp. **e** Maxilla. **f** Maxilla apex. **g** Maxilliped. **h** Article 3 of maxilliped. **i** Maxillule. **j** Maxillule apex. **k**–**o** Pleopods 1–5, respectively

***Male***. [New material SAMC-A091297; Figs. 5, 6.] Size 28.0 × 8.0. Body similar to females but much smaller and narrower; 3.1 times as long as wide. Pleopod 2 *appendix masculina* with parallel margins, 0.9 times as long as endopod, distally narrowly rounded.

**Remarks**

*Anilocra capensis* has a proportionally large ovoid and vaulted body with distinctly flexed distolateral margins of the pereonite 7. The pleotelson is wider than long and broadly rounded and the distal-most article of the antenna peduncle has one convex lateral margin. This species has the greatest range in proportional size of adult females and shows the most intrinsic variation with regards to pleonite width and uropod length. This was evident in the syntypes as well as the new fresh material examined. The freshly fixed specimens can be grouped mainly into two categories: specimens where pleonite 3 is most narrow and 5 is widest, and specimens where pleonite 1 and 2 are widest. Within each of these groups, proportional size and uropod length is variable, but no general differences in variability can be discerned between the two groups. There also appears to be colour variation (dark brown to red-brown, or tan) in *A. capensis*, but all the other characters to this species are observed in both colour types. Given that varying pleon morphology is the only character that can create groups within *A. capensis*, this is not sufficient to determine that there are two species.

In the present study *Anilocra capensis* was found attached to the skin of host fish. It was situated below the anterior portion of the dorsal fin, facing anteriorly, and above and not blocking the operculum. This attachment site was consistent among all the freshly collected material.

*Anilocra capensis* can be differentiated from the other species described below based on body, pereonite, pereopod and/or antenna/antennule form. The body shape of *A. capensis* is not twisted, whereas *Anilocra jovanasi* n. sp. and *Anilocra hadfieldae* n. sp. have weakly twisted bodies. Compared to *A. capensis*, *Anilocra paulsikkeli* n. sp. is 0.86 times as wide at the widest point and has notable sub-parallel body margins, and *Anilocra ianhudsoni* n. sp. is strongly narrowed anteriorly. *Anilocra capensis* can be distinguished from *Anilocra bunkleywilliamsae* n. sp., as the latter is proportionally shorter, pereonite 1 is longer, the antenna and antennule are narrower, and it has chromatophores. *Anilocra capensis* can be differentiated from *Anilocra angeladaviesae* n. sp., as *A. capensis* has a pronounced curvature on the lateral margin of the propodus of pereopod 6 that *A. angeladaviesae* n. sp. lacks. There is an elongated lateral margin of article 3 of the antenna of *A. angeladaviesae* n. sp. that extends onto article 4, and this does not occur in *A. capensis*.

We note that the 200-year-old female paralectotypes were similar in shape and form to the fresh material, but their poor and fragile condition, along with missing some articles made it possible to only include a few reliable drawings without further damage. The male paralectotype examined was significantly larger and likely in a different and later developmental stage than the freshly collected males. There was consistency in size and form of the fresh male material examined, but during our collections and those loaned from SAM, we found no male similar in size to Leach’s specimen.

It is important to note that the type-locality for *A. capensis* is Cape Town, South Africa, and the host is a sparid. Species of *Anilocra* are regarded as typically host specific to the family or genus level, and some even to the species level. Given this specificity and the general spatial distribution of *A. capensis* [31], it is unlikely that the records from the North Atlantic or other regions are of *A. capensis.* Thus, the reports of *A. capensis* from the Canary Islands and from hosts representing more than five families of fish are dubious [32, 33].

***Anilocra ianhudsoni*** **n. sp.**

***Type-host***: Unknown.

***Type-locality***: Nahoon River, East London, South Africa.

***Type-material***: Holotype: ♀ (18.0 × 8.0), paratypes: ♀ (18.0 × 8.0, dissected), ♂ (11.5 × 3.0, dissected) (SAMC-A091293). Collected by personnel on the ‘SS Pieter Faure’.

***Other-material***: Transitional (11.5 × 3.0) (SAMC-A091293).

***ZooBank registration***: The Life Science Identifier (LSID) for *Anilocra ianhudsoni* n. sp. is urn:lsid:zoobank.org:act:7AF506D2-5DD9-4B4A-8965-B1D224DE3650.

***Etymology***: This species is named for the first authorʼs (RLW) nephew, Ian Hudson Catalano, as a gesture of her appreciation to him and his mother, LM Catalano, for understanding the importance of her living abroad to conduct cymothoid-related research and to drive Ian’s curiosity in the natural sciences.

**Description**

**Fig. 7:**
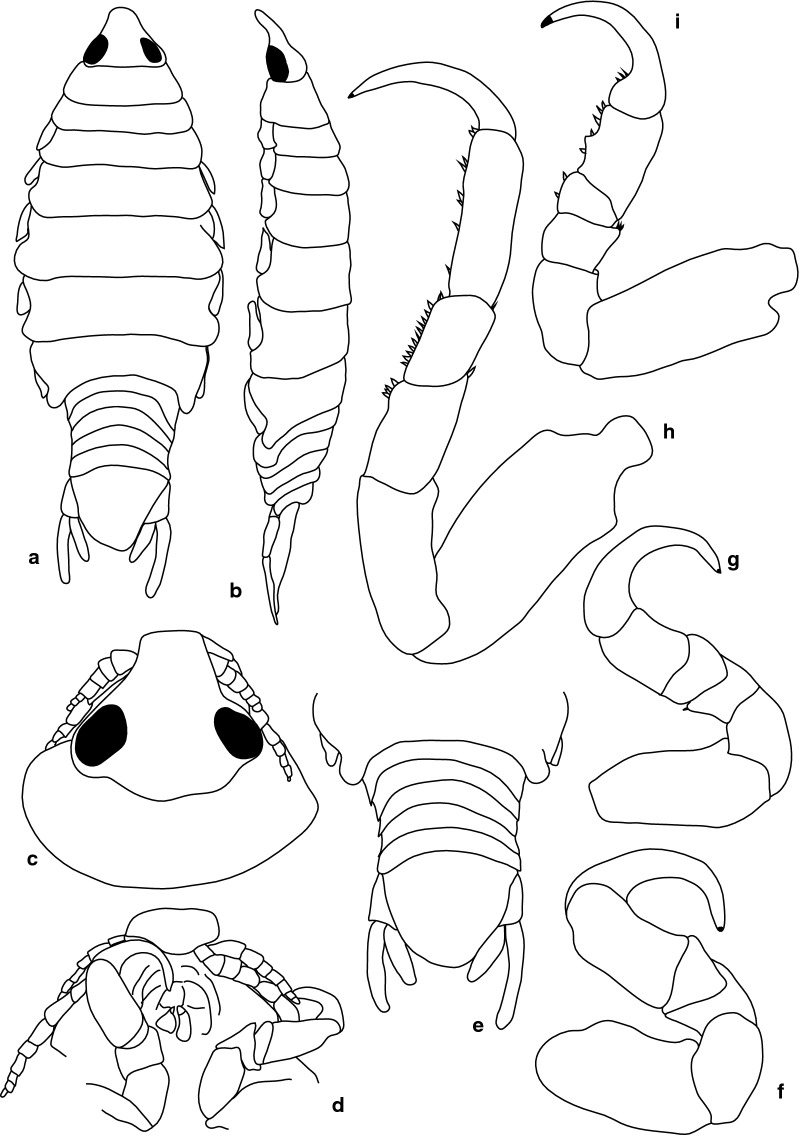
*Anilocra ianhudsoni* n. sp. ♀ (18.0 × 8.0, holotype SAMC-A091293). **a** Dorsal view. **b** Lateral view. **c** Dorsal view of cephalon. **d** Ventral view of cephalon. **e** Pleotelson (18.0 × 8.0, paratype SAM A6296). **f** Pereopod 1. **g** Pereopod 2. **h** Pereopod 6. **i** Pereopod 7

**Fig. 8:**
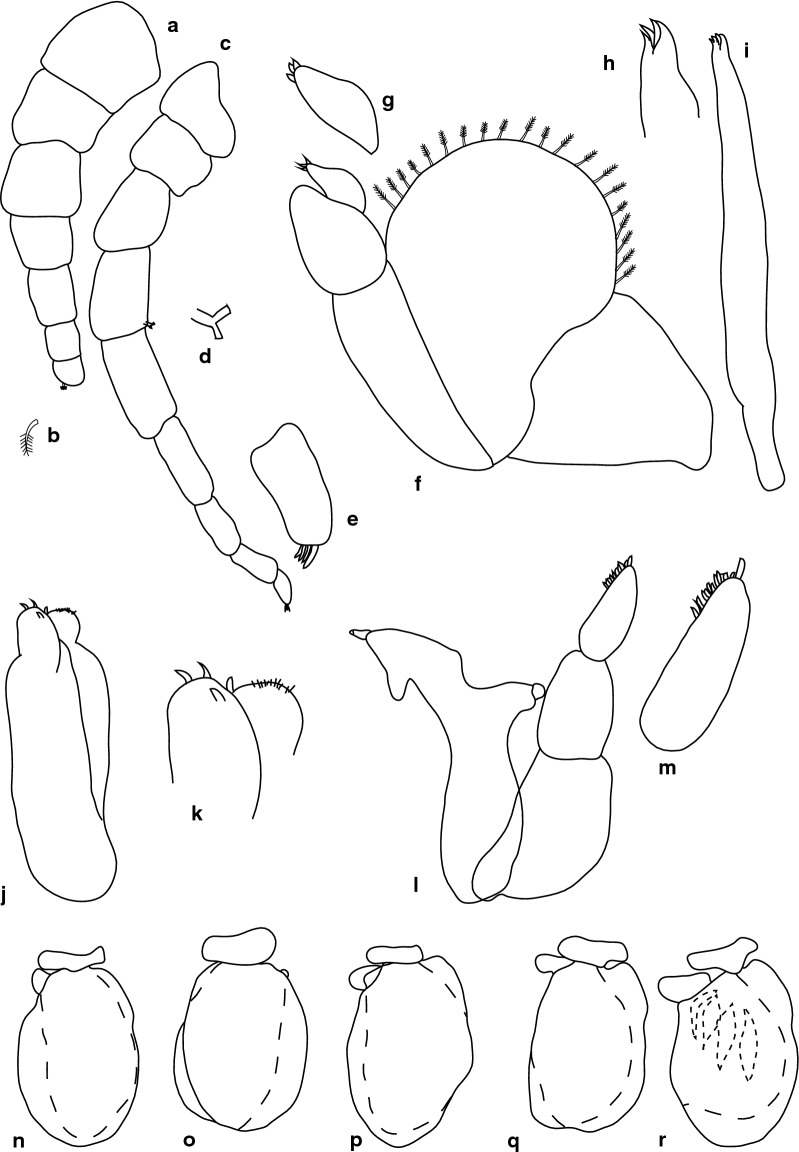
*Anilocra ianhudsoni* n. sp. ♀ (18.0 × 8.0, paratype SAMC-A091293). **a** Antennula. **b** Plumose setae of antennula terminal article. **c** Antenna. **d** Forked setae of antenna article 4. **e** Antenna terminal article. **f** Maxiliped. **g** Article 3 of maxiliped. **h** Maxillule apex. **i** Maxillule. **j** Maxilla. **k** Maxilla apex. **l** Mandible. **m** Article 3 of mandible. **n**–**r** Pleopods 1–5, respectively

***Female.*** [SAMC-A091293; Figs. 7, 8.] Size 18.0 × 8.0. Body diamond-shaped or ovoid, 2.6 times as long as greatest width; dorsal surfaces smooth and polished in appearance; widest at pereonite 5, most narrow at pereonite 1; pereonite lateral margins posteriorly protruding. Cephalon 0.9 as long as wide as measured in dorsal view, subtriangular. Eyes oval, with distinct margins, one eye width 0.3 times width of cephalon, one eye length 0.4 times length of cephalon. Pereonite 1 smooth, anterior border straight, anterolateral angle narrowly-rounded, in line with base of the cephalon. Posterior margins of pereonites smooth, slightly curved laterally. Coxae 2–3 wide, with posteroventral angles rounded; coxae 4–7 with rounded point and curved, extending past pereonite anterior margin. Pereonites 1–5 increasing in length and width; pereonites 6–7 decreasing in length and width; pereonites 5 and 6 subequal, pereonites 1–4 narrower, becoming more progressively rounded posteriorly. Pleon with pleonite 1 wider than pleonites 2–4, visible in dorsal view; posterior margin of pleonites 1–5 posteriorly concave, mostly concave. Pleonite 2 not overlapped by pereonite 7; posterolateral angles of pleonite 2 narrowly-rounded. Pleonite 1 similar in form to pleonites 2 and 3. Pleonites 3–5 similar in form to pleonite 2; pleonite 5 free, not overlapped by lateral margins of pleonite 4, posterior margin straight.

Pleotelson 0.9 times as long as anterior width, dorsal surface smooth, lateral margins convex, posterior margin converging to weak caudomedial point.

Antennula more stout than antenna; comprised of 7 articles; peduncle articles 1 and 2 distinct, articulated; article 2 0.6 times as long as article 1; article 3 1 times as long as wide, 0.5 times as long as combined lengths of articles 1 and 2; flagellum with 4 articles, extending to middle of eye, terminal article with 1 plumose seta. Antenna comprised of 9 articles; peduncle article 3 1.4 times as long as article 2; article 4 1.5 times as long as wide, 1.1 times as long as article 3; article 5 1.5 times as long as wide, 1.2 times as long as article 4; flagellum with 4 articles, terminal article terminating in 1–5 short simple setae, extending to middle of pereonite 1. Mandibular molar process ending in an acute incisor, with 13 simple setae. Maxillula simple with 4 terminal robust setae. Maxilla medial lobe partly fused to lateral lobe; 2 recurved robust setae; and 2 large recurved robust setae. Maxilliped weakly segmented, with lamellar oostegite lobe or second, smaller oostegite lobe on basal part of article, palp article 2 with 0 simple setae, article 3 with 3 recurved robust setae.

Pereopod 1 basis 1.7 times as long as greatest width; ischium 0.7 times as long as basis; merus proximal margin without bulbous protrusion; carpus with straight proximal margin; propodus 1.8 times as long as wide; dactylus moderately slender, 1.2 as long as propodus, 2.7 times as long as basal width. Pereopod 2 propodus 1.2 as long as wide; dactylus 1.9 as long as propodus. Pereopods gradually increasing in size towards posterior. Pereopod 6 basis 2.3 times as long as greatest width, ischium 0.5 times as long as basis, propodus 1.2 as long as wide, dactylus 1.8 as long as propodus. Pereopod 7 basis 2.8 times as long as greatest width; ischium 0.6 as long as basis, without protrusions; merus proximal margin without bulbous protrusion, merus 1.5 times as long as wide, 0.6 as long as ischium; carpus 1.7 times as long as wide, 0.5 as long as ischium, without bulbous protrusion; propodus 3.1 times as long as wide, 0.9 as long as ischium; dactylus slender, 1.0 as long as propodus, 4.5 times as long as basal width.

Pleopods without setae, exopod larger than endopod. Pleopod 1 exopod 0.7 times as long as wide, lateral margin weakly convex, distally narrowly rounded, medial margin weakly oblique, medial margin weakly convex; endopod 0.5 times as long as wide, lateral margin weakly convex, distally narrowly rounded, medial margin slightly convex, without retinaculae. Pleopods 2–5 similar to pleopod 1. Pleopods 3–5 endopods proximal borders do not extend below exopod to peduncle. Peduncle lobes absent.

Uropod longer than pleotelson, peduncle 0.5 times as long as rami, peduncle lateral margin without setae; rami extending beyond pleotelson, marginal setae absent, apices narrowly rounded. Endopod apically rounded, 3.0 times as long as greatest width, lateral margin weakly convex, medial margin weakly convex. Exopod extending beyond end of endopod, 6.0 times as long as greatest width, apically rounded, lateral margin weakly convex, medial margin weakly convex, terminating without setae.

**Fig. 9:**
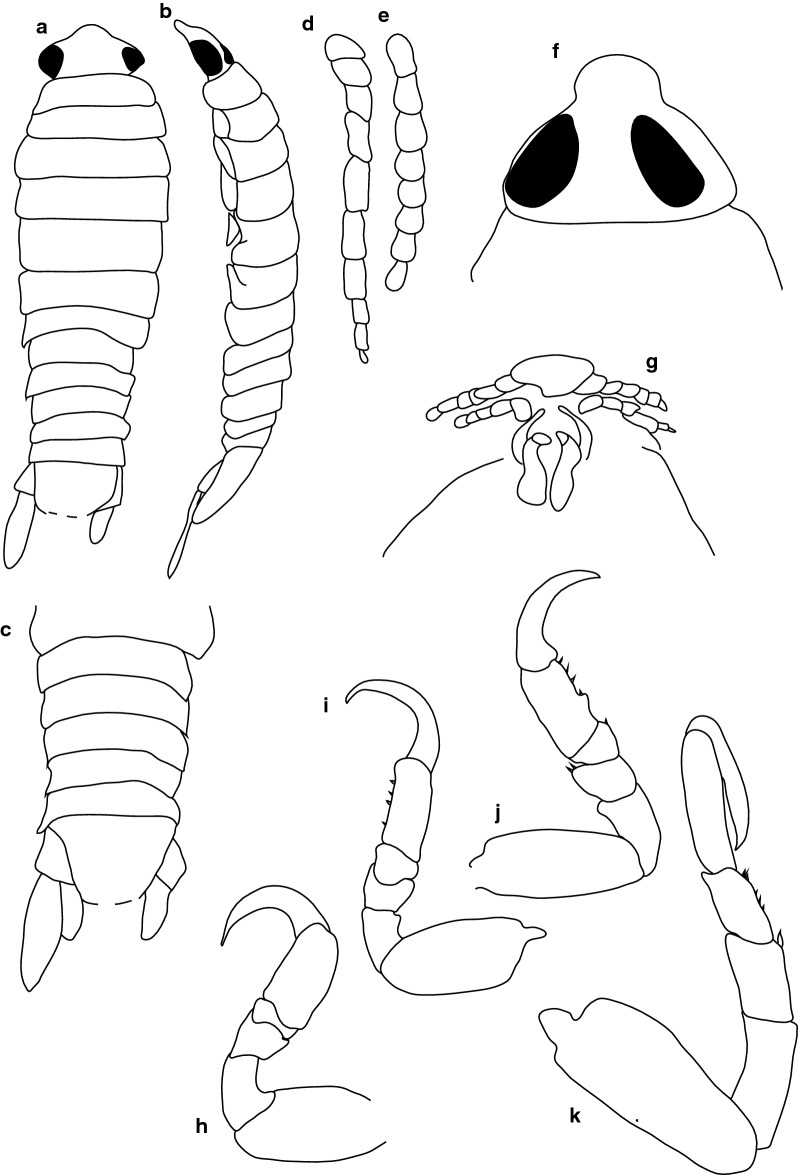
*Anilocra ianhudsoni* n. sp. ♂ (11.5 × 3.0, SAMC-A091293). **a** Dorsal view. **b** Lateral view. **c** Pleotelson. **d** Antenna. **e** Antennule. **f** Dorsal view of cephalon. **g** Ventral view of cephalon. **h** Pereopod 1. **i** Pereopod 2. **j** Pereopod 6. **k** Pereopod 7

***Male.*** [SAMC-A091293; Fig. 9.] Size 11.5 × 3.0. Smaller than female. Body rectangular, 3.4 times as long as wide. Pleopod 2 *appendix masculina* with parallel margins, distally narrowly rounded.

**Remarks**

*Anilocra ianhudsoni* n. sp. is among the smaller of the *Anilocra* species that occur in South Africa. The body shape is unique as it has a diamond-triangulate shape, such that pereonites 1–3 narrow strongly. Unlike the other African species, *A. ianhudsoni* n. sp. has no flexed pereonite posterolateral margins. The antennula has a plumose seta on the terminal article and a maxilla with simple setae or 3 recurved robust setae. *Anilocra ianhudsoni* n. sp. also has more slender pereopods and a flatter and broader antenna compared to the other species described herein.

*Anilocra ianhudsoni* n. sp. has a produced curvature on the lateral margin of the propodus of pereopod 6, which is similar to *A. capensis* and *A. bunkleywilliamsae* n. sp. Compared to *Anilocra ianhudsoni* n. sp., *A. capensis* and *A. bunkleywilliamsae* n. sp. both have broader, more ovoid pereonites, and a pleotelson that is closer in width to total body width.

***Anilocra bunkleywilliamsae*** **n. sp.**

***Type-host***: *Rhabdosargus holubi* Steindachner, 1881.

***Type-locality***: Mawalana Estuary, South Africa.

***Type-material***: Holotype ♀ (24.0 × 10.0) paratype ♀ (27.0 × 12.0, dissected) (SAMC-A091294). Collected by T. Mqolombia, on 17 February 1998.

***Other material***: Transitional (18.0 × 5.0).

***ZooBank registration***: The Life Science Identifier (LSID) for *Anilocra bunkleywilliamsae* n. sp. is urn:lsid:zoobank.org:act:9DF125DA-624A-4712-BA07-D45CF4BC7C8F.

***Etymology***: This species is named in honour of Dr Lucy Bunkley-Williams, a world expert on *Anilocra* who described nearly all of the known species of *Anilocra* from the Caribbean.

**Description**

**Fig. 10:**
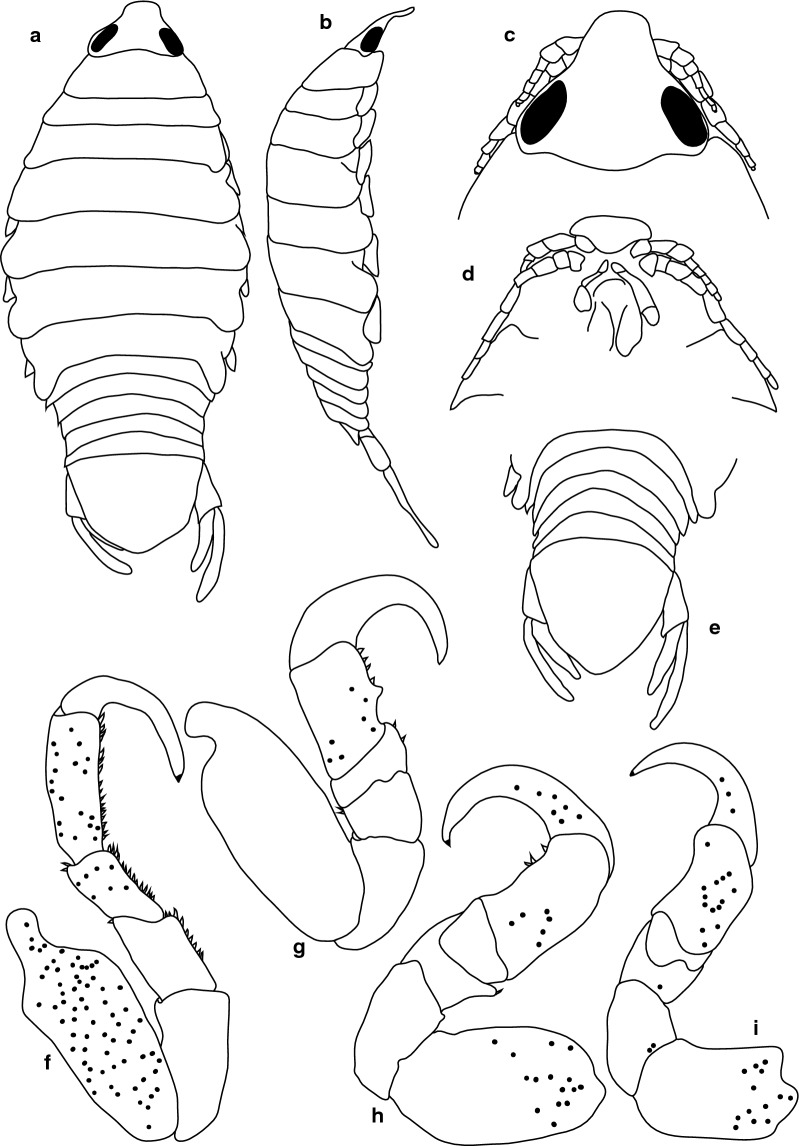
*Anilocra bunkleywilliamsae* n. sp. ♀ (24.0 × 10.0, holotype, SAMC-A091294). **a** Dorsal view. **b** Lateral view. **c** Dorsal view of cephalon. **d** Ventral view of cephalon. **e** Pleotelson (27.0 × 12.0, paratype SAMC-A091294). **f** Pereopod 7. **g** Pereopod 6. **h** Pereopod 2. **i** Pereopod 1

**Fig. 11:**
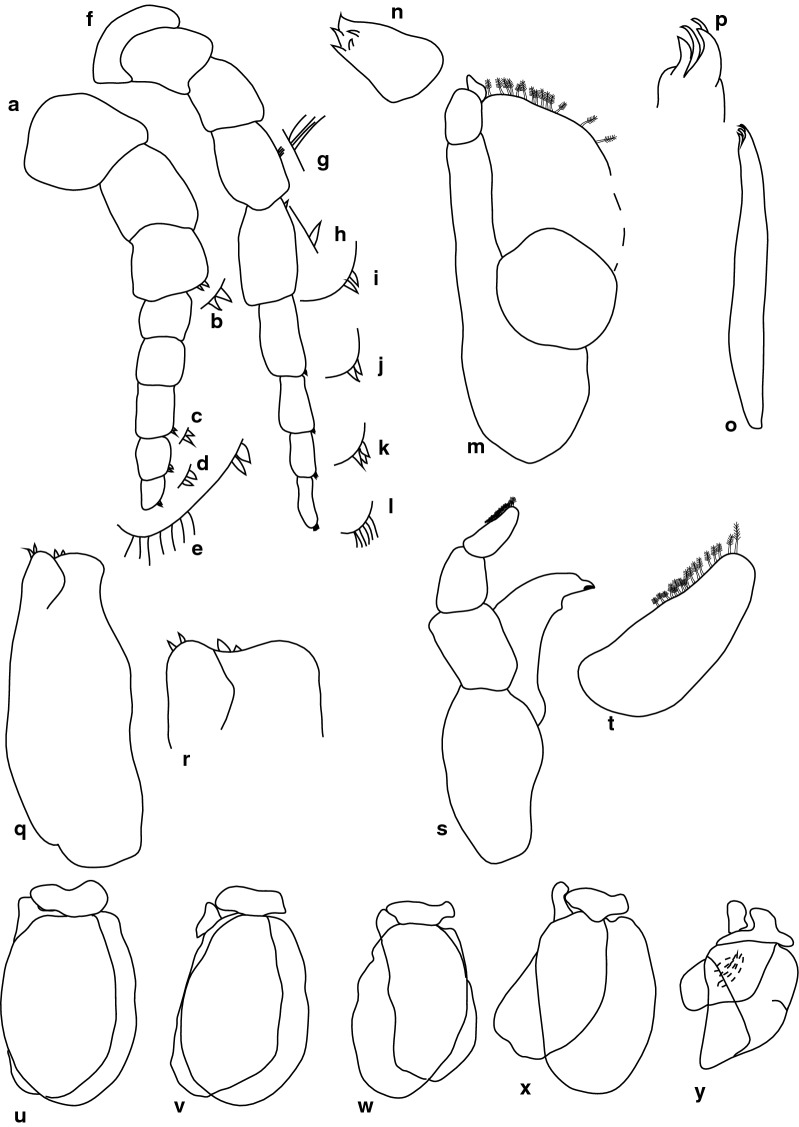
*Anilocra bunkleywilliamsae* n. sp. ♀ (27.0 × 12.0, paratype SAMC-A091294). **a**–**e** Antennula. **a** Antennula. **b** Setae of article 3. **c**–**e** Setae of articles 6–8, respectively. **f** Antenna. **g**–**l** Setae of antenna articles 4–9, respectively. **m** Maxilliped. **n** Article 3 of maxiliped. **o** Maxillule. **p** Maxillule apex. **q** Maxilla. **r** Maxilla apex. **s** Mandible. **t** Article 3 mandibular palp. **u**–**y** Pleopods 1–5, respectively

**Female.** [SAMC-A091294; Figs. 10, 11.] Size 27.0 × 12.0. Body 2.2 times as long as greatest width with dorsal surfaces smooth and polished in appearance. Body widest at pereonite 5, most narrow at pereonite 1, and pereonite lateral margins posteriorly protruding. Cephalon 0.8 as long as wide as measured in dorsal view, trapezoid-shaped. Eyes oval, with distinct margins, one eye width 0.2 times width of cephalon, one eye length 0.4 times length of cephalon. Pereonite 1 in line with base of cephalon, smooth, anterior border straight, anterolateral angle narrowly rounded. Posterior margins of pereonites smooth, straight. Coxae 2–3 narrow, with posteroventral angles rounded; coxae 4–7 with rounded point, not extending past posterior pereonite margin. Pereonites 1–5 increasing in length and width; pereonites 6–7 decreasing in length and width; pereonites 4–6 subequal, pereonites 1–3 subequal, increasing in similarity to pereonites 4–6. Pleon with pleonite 1 wider than pleonites 2–5, visible in dorsal view; posterior margin of pleonites 1–4 posteriorly concave, pleonite 5 posteriorly slightly concave to straight, mostly concave. Pleonite 2 not overlapped by pereonite 7; posterolateral angles of pleonite 2 narrowly rounded. Pleonite 1 similar in form to pleonites 2 and 3. Pleonites 3–5 similar in form to pleonite 2; pleonite 5 free, not overlapped by lateral margins of pleonite 4, posterior margin straight. Pleotelson 1 times as long as anterior width. Dorsal surface smooth. Pleotelson lateral margins convex, posterior margin converging to weak caudomedial point.

Antennula more stout than antenna; peduncle articles 1 and 2 distinct and articulated; article 2 0.8 times as long as article 1; article 3 1.0 times as long as wide, 0.4 times as long as combined lengths of articles 1 and 2; flagellum with 4 articles, extending to middle of eye, articles 3 and 6–8 with robust setae. Antenna comprised of 9 articles; peduncle article 3 1 times as long as article 2; article 4 0.7 times as long as wide, 0.9 times as long as article 3; article 5 1.6 times as long as wide, 1.5 times as long as article 4; flagellum with 4 articles, terminal article terminating in 6–10 short simple setae, extending to middle of pereonite 1. Mandibular molar process ending in an acute incisor, with 17 simple setae. Maxillula simple, with 4 terminal robust setae. Maxilla medial lobe partly fused to lateral lobe; lateral lobe with 0 simple setae, 2 recurved robust setae; medial lobe with 0 simple setae, and 2 large recurved robust setae. Maxilliped weakly segmented, with lamellar oostegite lobe or second, smaller oostegite lobe on basal part of article, palp article 2 with 0 simple setae, article 3 with 4 recurved robust setae. Oostegites margin covered in numerous plumose setae.

Pereopod 1 basis 1.7 times as long as greatest width; ischium 0.7 times as long as basis; merus proximal margin without bulbous protrusion; carpus with straight proximal margin; propodus 1.2 times as long as wide; dactylus moderately slender, 1.2 as long as propodus, 2.6 times as long as basal width. Pereopod 2 propodus 1.9 as long as wide; dactylus 1.1 as long as propodus. Pereopods gradually increasing in size towards posterior. Pereopod 6 basis 2.5 times as long as greatest width, ischium 0.4 times as long as basis, propodus 1.3 as long as wide, dactylus 1.4 as long as propodus. Pereopod 7 basis 2.8 times as long as greatest width; ischium 0.5 as long as basis, without protrusions; merus proximal margin without bulbous protrusion, merus 1.6 times as long as wide, 0.6 as long as ischium; carpus 2.0 times as long as wide, 0.6 as long as ischium, without bulbous protrusion; propodus 2.8 times as long as wide, 1 as long as ischium; dactylus slender, 1 as long as propodus, 3.6 times as long as basal width. Dense chromatophores present on 1–7.

Exopod larger than endopod. Pleopod 1 exopod 1.4 times as long as wide, lateral margin weakly convex, distally narrowly rounded, medial margin weakly oblique, medial margin weakly convex; endopod 1.6 times as long as wide, lateral margin weakly convex, distally narrowly rounded, medial margin slightly convex, peduncle 2.9 times as wide as long, without retinaculae. Pleopods 2–5 similar to pleopod 1. Pleopods 3–5 endopods proximal borders do not extend below exopod to peduncle. Peduncle lobes absent.

Uropod more than half the length of pleotelson, peduncle 0.5 times as long as rami, peduncle lateral margin without setae; rami extending beyond pleotelson, marginal setae absent, apices narrowly rounded. Endopod apically rounded, 5 times as long as greatest width, lateral margin weakly convex, medial margin weakly convex, terminating with 0 setae. Exopod extending beyond end of endopod, 9.7 times as long as greatest width, apically rounded, lateral margin weakly convex, medial margin weakly convex, terminating without setae.

**Remarks**

*Anilocra bunkleywilliamsae* n. sp. is most readily distinguished from all other African species as it has the most anteriorly rounded and arched rostrum, and more setae on the antennula and antenna of the species described herein. It also has large and dense chromatophores on its pereopods. The chromatophores are denser on the left side when in dorsal view.

Compared to *A. capensis*, the body of *A. bunkleywilliamsae* n. sp. is more ovoid, and pereopod 2 has robust setae present. Compared to *A. ianhudsoni* n. sp., *A. bunkleywilliamsae* n. sp. is generally smaller, its pleotelson converges more and is rounder, and its dactyls are shorter. The ovate and non-twisted body shape of *A. bunkleywilliamsae* n. sp. makes it distinguishable from *A. paulsikkeli* n. sp. which is long and narrow, and *A. jovanasi* n. sp. and *A. hadfieldae* n. sp. which are weakly twisted. *Anilocra bunkleywilliamsae* n. sp.is most similar in pereonite shape to *A. angeladaviesae* n. sp., but these species can be differentiated from each other as *A. angeladaviesae* n. sp. has no produced curvature on the lateral margin of the propodus of pereopod 6, its pereopods are more slender, and the general shape and form of the antenna and antennula are dissimilar.

***Anilocra paulsikkeli*** **n. sp.**

***Type-host***: Unknown.

***Type-locality***: Delagoa Bay, Mozambique.

***Type-material***: Holotype ♀ (32.0 × 8.5), paratypes: ♀ (33.0 × 9.0, dissected), ♂ (16.0 × 3.0, dissected; 18.0 × 4.0) (SAMC-A091295). Unknown collector.

***ZooBank registration***: The Life Science Identifier (LSID) for *Anilocra paulsikkeli* n. sp. is urn:lsid:zoobank.org:act:AFEA08B1-D27B-49F6-A30E-B4E142EFA5D9.

***Etymology***: This species is named in honour of Dr Paul C. Sikkel, the PhD supervisor of the first author (RLW) and research collaborator of the second author (NJS). Dr Sikkel’s devotion to furthering our knowledge on host-parasite interactions, strengthening the relationships between scientists and non-scientists, and rigorously mentoring future fish-parasite ecologists is hereby acknowledged.

**Description**

**Fig. 12:**
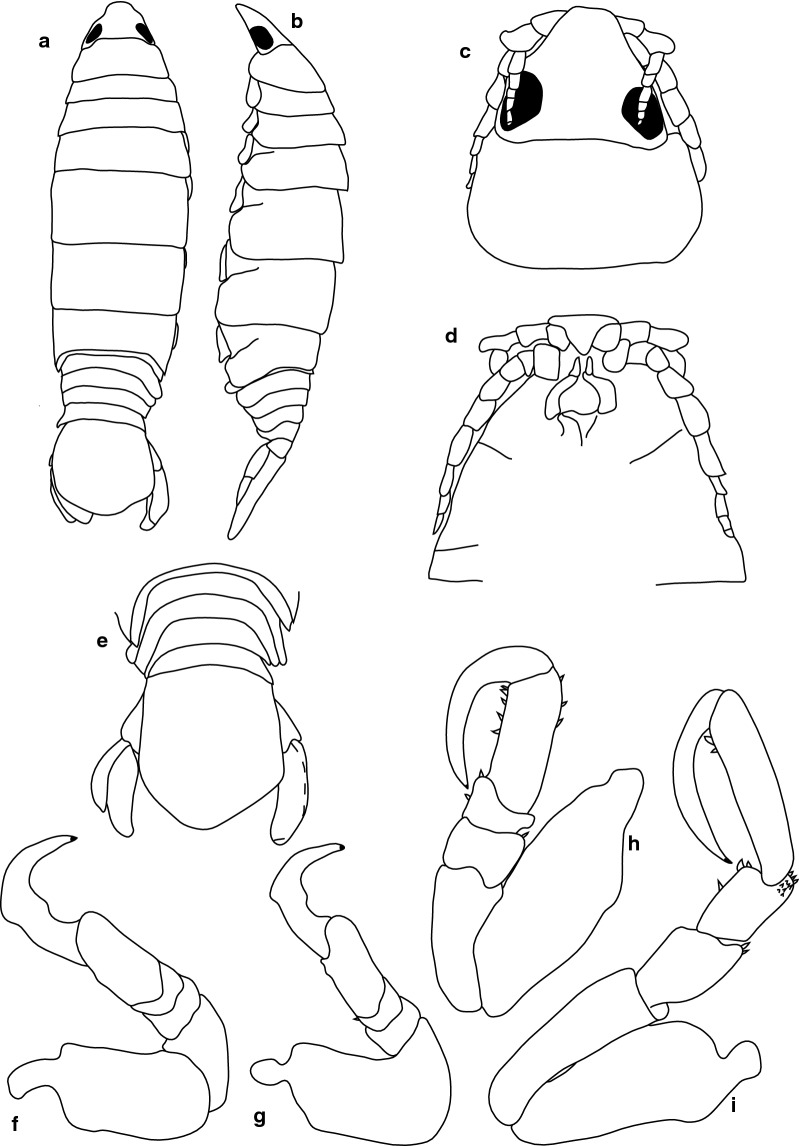
*Anilocra paulsikkeli* n. sp. ♀ (**a**–**e** 32.0 × 8.5, holotype, SAMC-A091295; **f–i** 33.0 × 9.0, paratype, SAMC-A091295). **a** Dorsal view. **b** Lateral view. **c** Dorsal view of cephalon. **d** Ventral view of cephalon. **e** Pleotelson. **f** Pereopod 1. **g** Pereopod 2. **h** Pereopod 6. **i** Pereopod 7

**Fig. 13:**
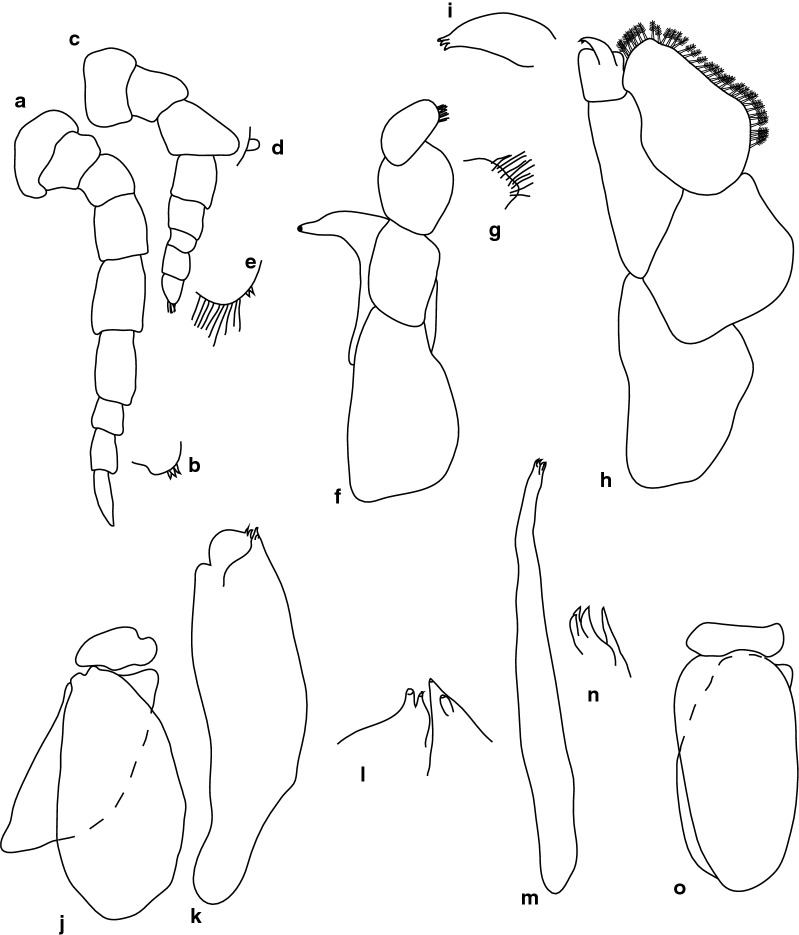
*Anilocra paulsikkeli* n. sp. ♀ (33.0 × 9.0, paratype, SAMC-A091295). **a**–**b** Antenna. **a** Antenna. **b** Setae of article 8. **c**–**e** Antennula. **c** Antennula. **d** Setae of article 3. **e** Setae of terminal article. **f** Mandible. **g** Article 3 mandibular palp. **h** Maxilliped. **i** Article 3 of maxilliped. **j** Pleopod 1. **k** Maxilla. **l** Maxilla apex. **m** Maxillule. **n** Maxillule apex. **o** Pleopod 2

***Female.*** [SAMC-A091295; Figs. 12–13.] Size 33.0 × 9.0. Body rectangular or elongate, 3.7 times as long as greatest width, dorsal surfaces smooth and polished in appearance, widest at pereonite 5, most narrow at pereonite 1, pereonite lateral margins subparallel. Cephalon 0.73 as long as wide as measured in dorsal view, trapezoid-shaped. Eyes oval, with distinct margins, one eye width 0.2 times width of cephalon; one eye length 0.45 times length of cephalon. Pereonite 1 in line with base of cephalon, smooth, anterior border straight, anterolateral angle narrowly rounded. Posterior margins of pereonites smooth and straight. Coxae 2–3 wide; with posteroventral angles rounded; 4–7 with rounded point and curved; not extending past posterior pereonite margin. Pereonites 1–4 increasing in length and width; pereonites 6–7 decreasing in length and width; pereonites 5 and 6 subequal. Pleon with pleonite 1 largely concealed by pereonite 7, visible in dorsal view; pleonites posterior margin 1–4 posteriorly concave, pleonite 5 posteriorly slightly concave to straight, mostly concave. Pleonite 2 not overlapped by pereonite 7; posterolateral angles of pleonite 2 expanded, posteriorly produced or rounded. Pleonite 1 differ in form to pleonite 2 and 3. Pleonite 5 free, not overlapped by lateral margins of pleonite 4, with posterolateral angles narrowly rounded, posterior margin straight. Pleotelson 1.31 times as long as anterior width. Dorsal surface smooth. Pleotelson lateral margins convex, posterior margin converging to weak caudomedial point.

Antennula approximately as stout as antenna, comprised of 9 articles; peduncle articles 1 and 2 distinct and articulated; article 2 1.3 times as long as article 1; article 3 0.9 times as long as wide, 0.6 times as long as combined lengths of articles 1 and 2; flagellum with 5 articles, terminal article with robust and simple setae, extending to posterior margin of eye. Antenna comprised of 9 articles; peduncle article 3 1.0 times as long as article 2; article 4 1.15 times as long as wide, 1.5 times as long as article 3; article 5 1.4 times as long as wide, 1.2 times as long as article 4; flagellum with 4 articles, terminal article terminating in no setae, extending to middle of pereonite 1. Mandibular molar process ending in an acute incisor, with 11 simple setae. Maxillula simple, with 2 terminal robust setae. Maxilla medial lobe partly fused to lateral lobe; 2 recurved robust setae and 2 large recurved robust setae. Maxilliped weakly segmented, with lamellar oostegite lobe or second, smaller oostegite lobe on basal part of article, article 3 with 3 recurved robust setae. Oostegites margin covered in numerous plumose setae.

Pereopod 1 basis 2.5 times as long as greatest width; ischium 0.5 times as long as basis; merus proximal margin with slight bulbous protrusion; carpus with straight proximal margin; propodus 2.1 times as long as wide; dactylus stout, 1.2 as long as propodus, 3 times as long as basal width. Pereopod 2 propodus 2.5 as long as wide; dactylus 1.1 as long as propodus. Pereopods gradually increasing in size towards posterior. Pereopod 6 basis 2.7 times as long as greatest width, ischium 0.5 times as long as basis, propodus 2.6 as long as wide, dactylus 1 as long as propodus. Pereopod 7 basis 2.4 times as long as greatest width; ischium 0.7 as long as basis, without protrusions; merus proximal margin without bulbous protrusion, merus 1.7 times as long as wide, 0.6 as long as ischium; carpus 1.5 times as long as wide, 0.44 as long as ischium, without bulbous protrusion; propodus 4.3 times as long as wide, 1.1 as long as ischium; dactylus slender, 0.9 as long as propodus, 4.4 times as long as basal width.

Pleopods without setae, exopod larger than endopod. Pleopod 1 exopod 2.1 times as long as wide, lateral margin weakly convex, distally narrowly rounded, medial margin weakly oblique, medial margin weakly convex; endopod 2.2 times as long as wide, lateral margin weakly convex, distally narrowly rounded, medial margin slightly convex, peduncle 2.0 times as wide as long, without retinaculae. Pleopods 3–5 endopods proximal borders do not extend below exopod to peduncle. Peduncle lobes absent. Uropod more than half the length of pleotelson, peduncle 0.7 times as long as rami, lateral margin without setae; rami extending to pleotelson apex, marginal setae absent, apices broadly rounded. Endopod apically rounded, 3.3 times as long as greatest width, lateral margin weakly convex, medial margin weakly convex, terminating with 0 setae. Exopod not extending to end of endopod, 6.7 times as long as greatest width, apically rounded, lateral margin weakly convex, medial margin weakly convex, terminating without setae.

**Fig. 14:**
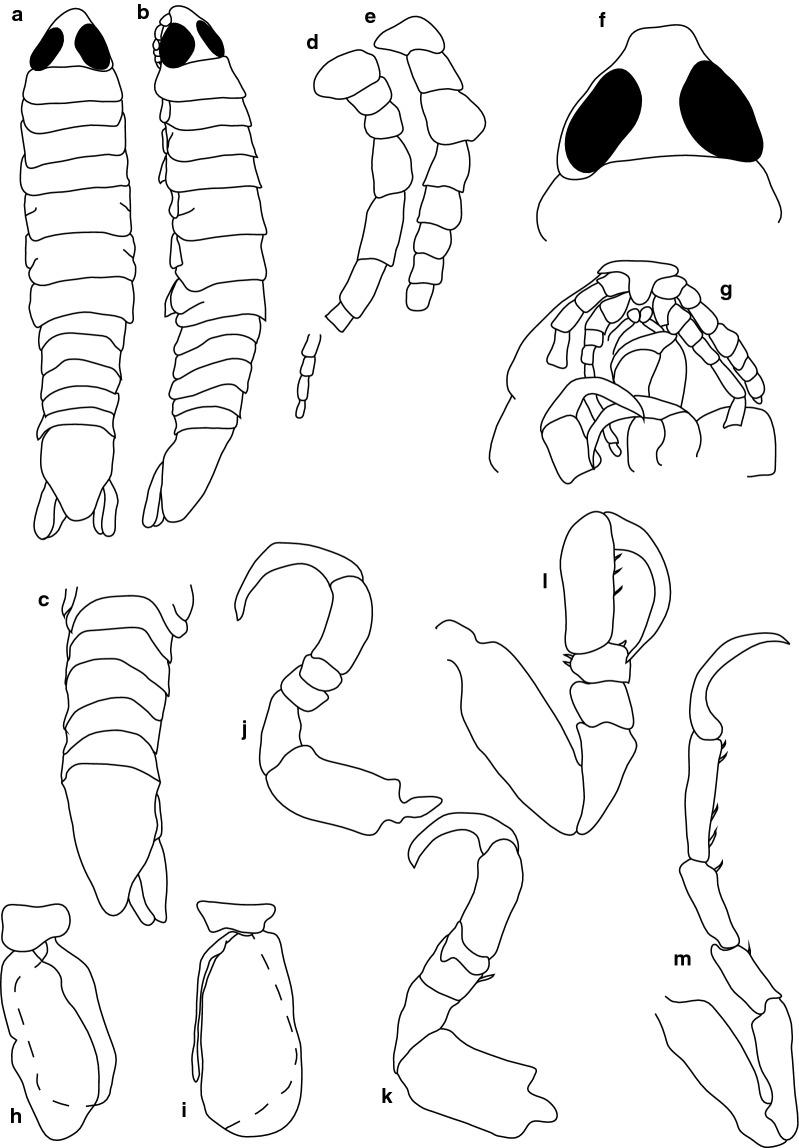
*Anilocra paulsikkeli* n. sp. ♂ (16.0 × 3.0, SAMC-A091295). **a** Dorsal view. **b** Lateral view. **c** Pleotelson. **d** Antenna, damaged. **e** Antennula. **f** Dorsal view of cephalon. **g** Ventral view of cephalon. **h** Pleopod 1. **i** Pleopod 2. **j** Pereopod 1. **k** Pereopod 2. **l** Pereopod 6. **m** Pereopod 7

***Male.*** [SAMC-A091295; Fig. 14.] Size 16.0 × 3.0. Similar to female but much smaller. Body rectangular, weakly twisted, 4.9 times as long as wide. Pleopod 2 *appendix masculina* with parallel margins, 0.8 times as long as endopod, distally narrowly rounded.

**Remarks**

*Anilocra paulsikkeli* n. sp. has a distinctive elongate, narrow body, with sub-parallel lateral margins. Pleonite 1 is partly covered by pereonite 7. The lateral margins of the pleotelson are sub-parallel and from the last third of the pleotelson there is a strong convergence to a rounded medial point. The eyes of *A. paulsikkeli* n. sp. are nearly one-quarter the width of the cephalon.

*Anilocra paulsikkeli* n. sp. is similar to *A. leptosoma*, but there are multiple differences*. Anilocra paulsikkeli* n. sp. is larger (3.7 times as long as wide whereas *A. leptosoma* is 3.4 times as long as wide), the first antenna article 3 is not produced, pleonite 1 is more concealed, and the posterior margin of the pleonites are straighter. Aneesh et al. [34] recently resdescribed *A. leptosoma* but did not examine the *A. leptosoma* lectotype material, and the drawings of Aneesh are in disagreement with Bruce 1987’s drawings of the lectotype. In Aneesh et al. [34], Fig. 1d, e appear to not resemble *A. leptosoma*, and are more similar to *A. capensis*, with respect to body shape and form in dorsal view. Aneesh et al. [34] likely did not identify *A. leptosoma.* To identify the species these authors report on, specimens should be further examined morphologically, and molecular data should be provided.

*Anilocra paulsikkeli* n. sp. is also similar to *Anilocra caudata* Bovallius, 1887 but can be distinguished from *A. caudata*, by the wider pleotelson, pleonite 1 not being covered by pereonite 7 and the lateral margins of pleonite 5 are more acute.

Compared to the other species described herein, *A. paulsikkeli* n. sp. and *A. jovanasi* n. sp. are most similar. Both species have nodules that occur midway on the lateral margins of the dactyls of pereopods 1 and 2, but the nodules are larger on *A. paulsikkeli* n. sp. *Anilocra jovanasi* n. sp. also has antennula peduncle article 3 more strongly produced than that of *A. paulsikkel* n. sp.

***Anilocra jovanasi*** **n. sp.**

***Type-host***: Unknown.

***Type-locality***: Delagoa Bay, Mozambique.

***Type-material***: Holotype ♀ (20.0 × 7.0, dissected) (SAMC-A091296). Unknown collector.

***ZooBank registration***: The Life Science Identifier (LSID) for *Anilocra jovanasi* n. sp. is urn:lsid:zoobank.org:act:DA044D6D-CA90-4E20-9D76-1D6FD636569C.

***Etymology***: This species is named for Professor Jo G. van As (1949–2018), the late aquatic parasitologist and PhD advisor of the second author (NJS). Professor van As revived the field of aquatic parasitology in South Africa in the 1970s and all current active aquatic parasitologists in South Africa are either his former students, or a student of one of his former students. His contribution to the field of aquatic parasitology in South Africa is hereby acknowledged.

**Description**

**Fig. 15:**
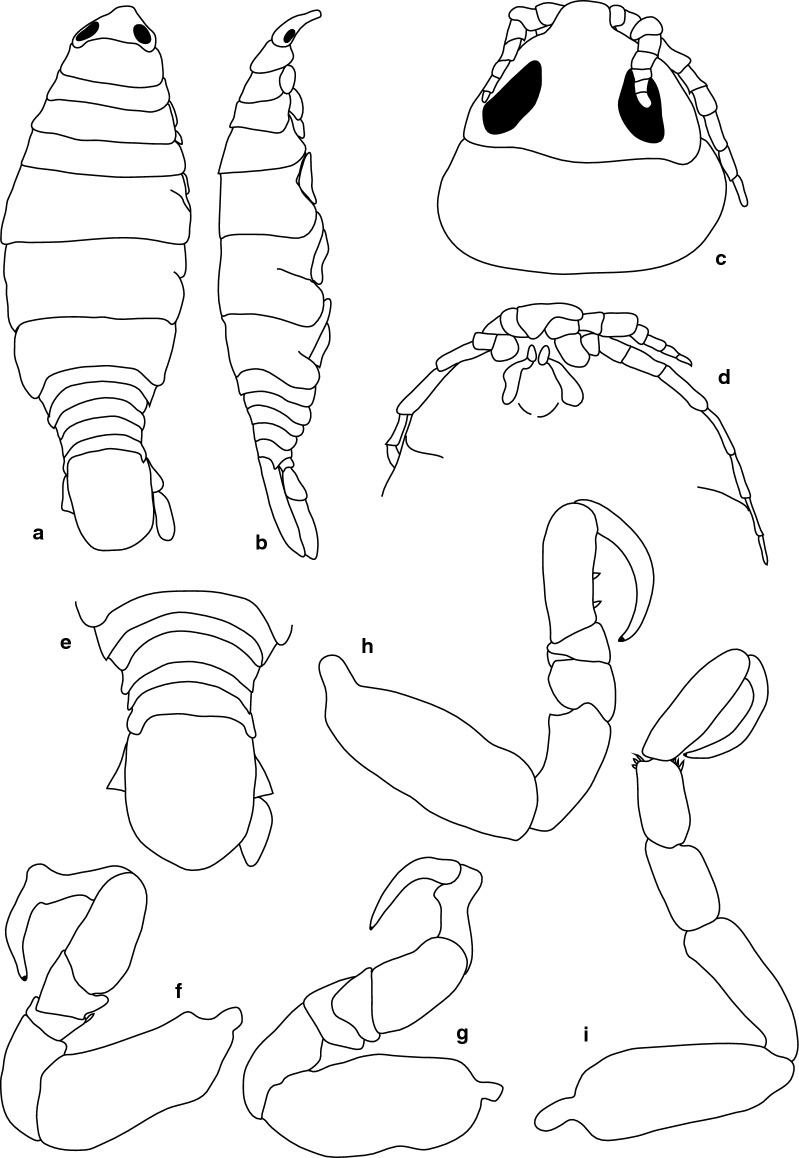
*Anilocra jovanasi* n. sp. ♀ (20.0 × 7.0, holotype, SAMC-A091296). **a** Dorsal view. **b** Lateral view. **c** Dorsal view of cephalon. **d** Ventral view of cephalon. **e** Pleotelson. **f** Pereopod 1. **g** Pereopod 2. **h** Pereopod 6. **i** Pereopod 7

**Fig. 16:**
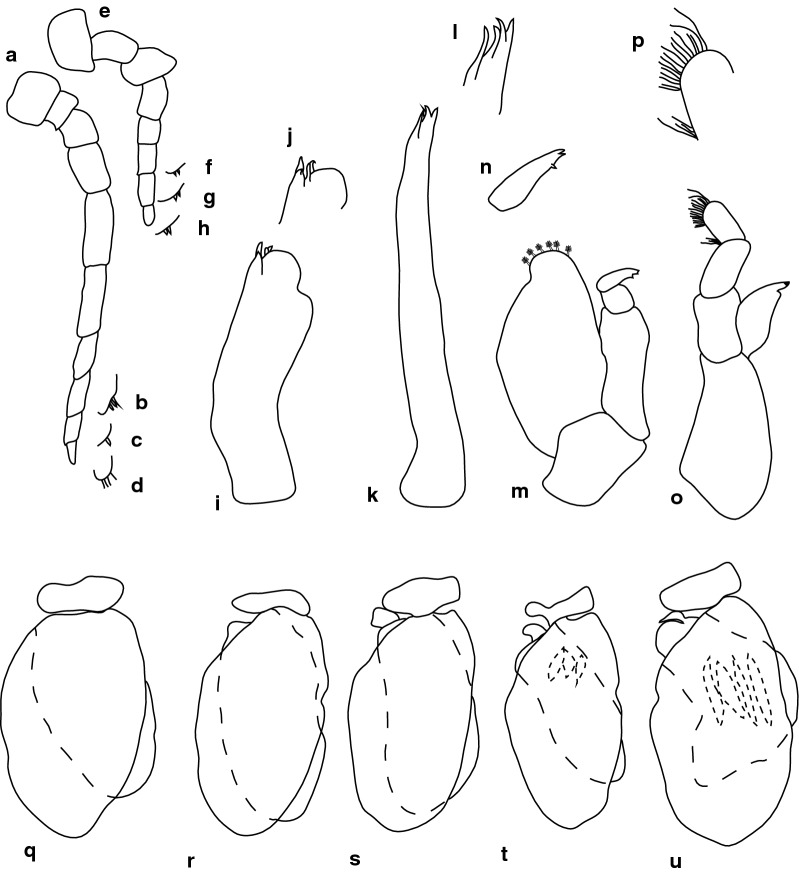
*Anilocra jovanasi* n. sp. ♀ (20.0 × 7.0, holotype, SAMC-A091296). **a**–**d** Antenna. **a** Antenna. **b**–**d** Setae of articles 8–10, respectively. **e**–**h** Antennula. **e** Antennula. **f**–**h** Setae of articles 6–8, respectively. **i** Maxilla. **j** Maxilla apex. **k** Maxillule. **l** Maxillule apex. **m** Maxilliped. **n** Article 3 of maxilliped. **o** Mandible. **p** Mandibular palp. **q**–**u** Pleopods 1–5, respectively

***Female***. [SAMC-A091296; Figs. 15, 16.] Size 20.0 × 7.0. Body ovoid and weakly twisted, 2.9 times as long as greatest width, dorsal surfaces smooth and polished in appearance, most narrow at pereonite 1, pereonite lateral margins mostly posteriorly ovate. Cephalon 0.67 as long as wide as measured in dorsal view, trapezoid-shaped. Eyes oval, with distinct margins, one eye width 0.2 times width of cephalon; one eye length 0.4 times length of cephalon. Pereonite 1 in line with base of cephalon, smooth, anterior border straight, anterolateral angle narrowly rounded. Posterior margins of pereonites smooth and straight, 5 extended posteriorly and overlay lateral margins of pleotelson. Coxae 2–3 wide; with posteroventral angles rounded; coxae 4–7 with rounded point and curved; not extending past posterior pereonite margin. Pereonites 1–5 increasing in length and width; pereonites 6–7 decreasing in length and width; pereonites 5 and 6 subequal, pereonites 1–4 narrower. Pleon with pleonite 1 wider than 2–4, visible in dorsal view; posterior margin of pleonites 1–4 posteriorly concave, pleonite 5 posteriorly slightly concave to straight, mostly concave. Pleonite 2 not overlapped by pereonite 7; posterolateral angles of pleonite 2 narrowly rounded. Pleonite 1 similar in form to pleonite 2. Pleonites 3–5 differ in form from pleonite 5; pleonite 5 free, not overlapped by lateral margins of pleonite 4, posterolateral margins extend onto anterior portion of pleotelson, posterior margin straight. Pleotelson 1.3 times as long as anterior width, dorsal surface smooth, lateral margins weakly convex, posterior margin converging to weak caudomedial point.

Antennula approximately as stout as antenna; comprised of 8 articles; peduncle articles 1 and 2 distinct and articulated; article 2 1.1 times as long as article 1; article 3 0.6 times as long as wide, 0.4 times as long as combined lengths of articles 1 and 2; flagellum with 5 articles, articles 6–8 with robust setae, extending to middle of eye. Antenna comprised of 10 articles; peduncle article 3 1.7 times as long as article 2; article 4 1.4 times as long as wide, 1.4 times as long as article 3; article 5 2.6 times as long as wide, 1.5 times as long as article 4; flagellum with 5 articles, articles 8–9 with setae, terminal article terminating in 1–5 short simple setae, extending to middle of pereonite 1. Mandibular molar process present, with 20 simple setae. Maxilla medial lobe partly fused to lateral lobe; 2 recurved robust setae; and 2 large recurved robust setae. Maxilliped weakly segmented, with lamellar oostegite lobe, palp article 2 with 0 simple setae, article 3 with 3 recurved robust setae. Oostegites margin covered in numerous plumose setae.

Pereopod 1 basis 2.7 times as long as greatest width; ischium 0.6 times as long as basis; merus proximal margin without bulbous protrusion; carpus with straight proximal margin; propodus 2 times as long as wide; dactylus slender, 1.2 as long as propodus, 3.2 times as long as basal width. Pereopod 2 propodus 2.15 as long as wide; dactylus 1 as long as propodus. Pereopods gradually increasing in size towards posterior. Pereopod 6 basis 2.91 times as long as greatest width, ischium 0.5 times as long as basis, propodus 2.6 as long as wide, dactylus 1.1 as long as propodus. Pereopod 7 basis 3.1 times as long as greatest width; ischium 0.6 as long as basis, without protrusions; merus proximal margin without bulbous protrusion, merus 2.0 times as long as wide, 0.7 as long as ischium; carpus 1.9 times as long as wide, 0.6 as long as ischium, without bulbous protrusion; propodus 3.2 times as long as wide, 0.9 as long as ischium; dactylus slender, 0.86 as long as propodus, 5.0 times as long as basal width.

Pleopods without setae, exopod larger than endopod. Pleopod 1 exopod 1.6 times as long as wide, lateral margin weakly convex, distally narrowly rounded, medial margin weakly oblique, medial margin weakly convex; endopod 1.6 times as long as wide, lateral margin weakly convex, distally narrowly rounded, medial margin slightly convex, peduncle 2.4 times as wide as long, without retinaculae. Pleopods 2–5 similar to pleopod 1. Pleopods 3–5 endopods proximal borders do not extend below exopod to peduncle. Peduncle lobes present on 5.

Uropod more than half the length of pleotelson, peduncle lateral margin without setae; rami not extending beyond pleotelson, marginal setae absent, apices narrowly rounded. Endopod apically rounded, lateral margin weakly convex, medial margin weakly convex. Apically rounded, lateral margin weakly convex, medial margin weakly convex.

**Remarks**

The body *Anilocra jovanasi* n. sp. is weakly twisted and narrowly ovoid. In dorsal view, the antennula appears geniculate due to the anterodistal lateral angle of the third article; pereopods 6 and 7 possess few robust setae. *Anilocra jovanasi* n. sp. is the only species described in southern Africa with the posterolateral margins of the fifth pleonite extending onto the dorsal portion of the pleotelson.

*Anilocra jovanasi* n. sp. is most similar in body shape to *A. leptosoma*, and *A. paulsikkeli* n. sp. However, the body of *A. leptosoma* is not twisted and the pleotelson converges strongly to a distinct point, whereas in *A. jovanasi* n. sp., the body is straight and the pleotelson converges broadly to a very weak point. The pleotelsons of *A. paulsikkeli* n. sp. and *A. jovanasi* n. sp. are similar in shape until the last third, at which point the lateral margins of the pleotelson of *A. jovanasi* n. sp. converge broadly to a very weak point, and those of *A. paulsikkeli* n. sp. converge strongly to a broad medial point. Moreover, pleonite 1 of *A. jovanasi* n. sp. is substantially more visible than that of *A. paulsikkeli* n. sp. Whereas *A. paulsikkeli* n. sp. has distinct nodules on the dactyls of pereopods 1 and 2, this is present only on pereopod 1 of *A. jovanasi* n. sp. The propodus of pereopod 2 is without a curved produced curvature on the lateral margin, separating it from *A. capensis* and *A. bunkleywilliamsae* n. sp. *Anilocra ianhudsoni* n. sp. has a distinctly different body form and its pereopods 6 and 7 are more dense in robust setae. *Anilocra angeladaviesae* n. sp. is more robustly ovoid, with a broader pleotelson than *A. jovanasi* n. sp. *Anilocra hadfieldae* n. sp. also has a weakly twisted body, but compared to *A. jovanasi* n. sp., it has a more produced pointed lateral margin of pleonite 7 and a medial indent on the pleotelson.

***Anilocra angeladaviesae*** **n. sp.**

Syn. *Anilocra capensis* Monod, 1924

***Type-host***: Uncertain (see Remarks).

***Type-locality***: Cape Blanc, Morocco.

***Type-material***: Holotype ♀ (BMNH1924.5.30.6) (37.0 × 18.0), paratypes (BMNH1924.5.30.7–10): ♀ (38.0 × 17.0; 37.0 × 15.0; 35.0 × 13.0), ♂ (24.0 × 6.0; 18.0 × 5.0). There are 6 accession numbers but 7 specimens in this lot. Collected by M. Theo Monod, 1923.

***ZooBank registration***: The Life Science Identifier (LSID) for *Anilocra angeladaviesae* n. sp. is urn:lsid:zoobank.org:act:3381D22E-67B0-4847-A559-0899B20E9F7D.

***Etymology***: This species is named after Professor Angela Josephine Davies (1947–2013), PhD promotor and postdoctoral advisor to the second author (NJS), to commemorate her contribution to the knowledge of crustacean parasites of fishes, as well as her singular dedication to- and enthusiasm for sharing this knowledge with all those she mentored.

**Description**

**Fig. 17:**
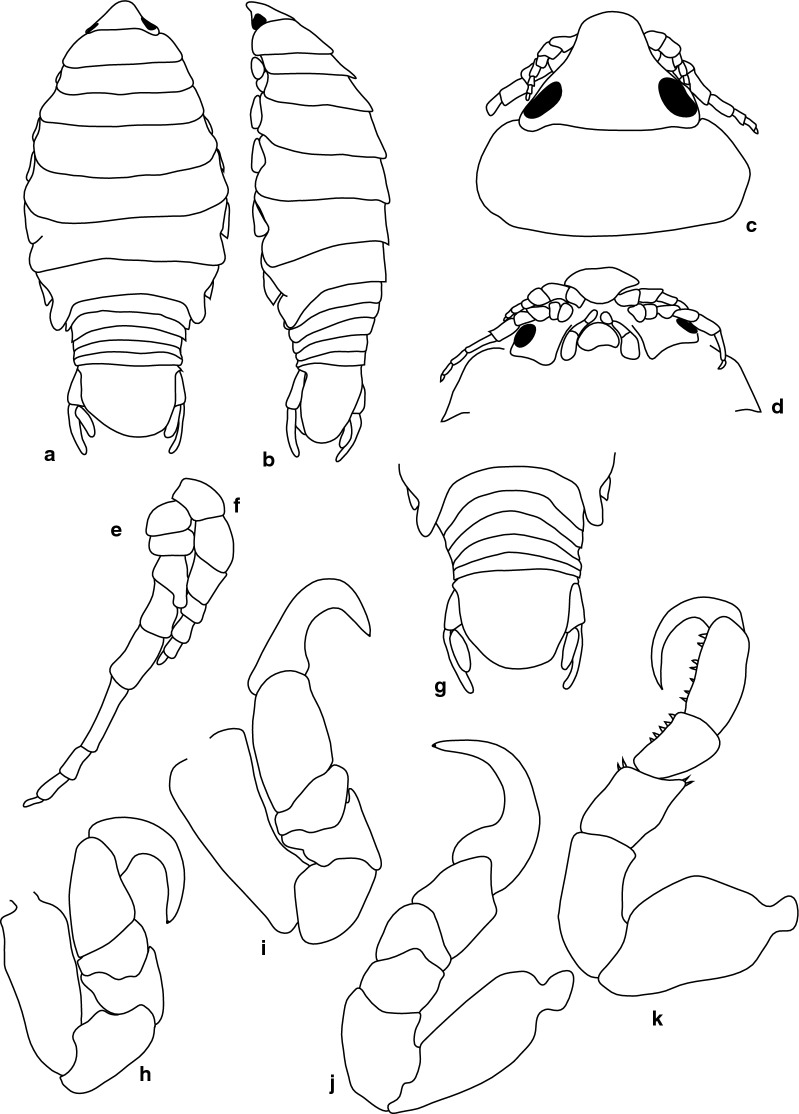
*Anilocra angeldaviesae* n. sp. ♀ (37.0 × 18.0, holotype, BMNH1924.5.30.6). **a** Dorsal view. **b** Lateral view. **c** Dorsal view of cephalon. **d** Ventral view of cephalon. **e** Antenna. **f** Antennula. **g** Pleotelson. **h** Pereopod 1. **i** Pereopod 2. **j** Pereopod 6. **k** Pereopod 7

***Female***. [BMNH1924.5.30.6; Fig. 17.] Size 37 × 18. Body ovoid, 2.1 times as long as greatest width, dorsal surfaces smooth and polished in appearance, widest at pereonite 5, most narrow at pereonite 1, pereonite lateral margins mostly posteriorly ovate. Cephalon 0.7 as long as wide as measured in dorsal view, trapezoid-shaped. Eyes oval, with distinct margins, one eye width 0.7 times width of cephalon; one eye length 0.3 times length of cephalon. Pereonite 1 in line with base of cephalon, smooth, anterior border straight, anterolateral angle narrowly rounded. Posterior margins of pereonites smooth and straight. Coxae 2–3 wide; with posteroventral angles rounded; coxae 4–7 with rounded point and curved; not extending past posterior pereonite margin. Pereonites 1–5 increasing in length and width; pereonites 6–7 decreasing in length and width; pereonites 1–5 subequal, pereonites 6–7 subequal. Pleon with pleonite 1 wider than pleonites 2–4, visible in dorsal view; posterior margin of pleonites 1–4 posteriorly concave, pleonite 5 posteriorly slightly concave to straight, mostly concave. Pleonite 2 not overlapped by pereonite 7; posterolateral angles of pleonite 2 narrowly rounded. Pleonite 1 similar in form to pleonite 2. Pleonites 3–5 similar in form to pleonite 2; pleonite 5 free, not overlapped by lateral margins of pleonite 4, posterior margin straight. Pleotelson 0.8 times as long as anterior width, dorsal surface smooth, lateral margins weakly convex, posterior margin evenly rounded.

Antennula more stout than antenna, comprised of 7 articles; peduncle articles 1 and 2 distinct and articulated; article 2 1.2 times as long as article 1; article 3 1.1 times as long as wide, 0.4 times as long as combined lengths of articles 1 and 2; flagellum with 4 articles, extending to middle of eye. Antenna comprised of 9 articles. Peduncle article 3 2.17 times as long as article 2; article 4 1.5 times as long as wide, 0.9 times as long as article 3; article 5 2.0 times as long as wide, 1.3 times as long as article 4. Antenna flagellum with 4 articles, extending to middle of pereonite 1.

Pereopod 1 basis 2.8 times as long as greatest width; ischium 1.9 times as long as basis; merus proximal margin without bulbous protrusion; carpus with straight proximal margin; propodus 1.8 times as long as wide; dactylus stout, 1.1 as long as propodus, 2.3 times as long as basal width. Pereopod 2 propodus 2.3 as long as wide; dactylus 0.9 as long as propodus. Pereopods gradually increasing in size towards posterior. Pereopod 6 basis 3 times as long as greatest width, ischium 0.6 times as long as basis, propodus 1.2 as long as wide, dactylus 2 as long as propodus. Pereopod 7 basis 2.0 times as long as greatest width; ischium 0.7 as long as basis, without protrusions; merus proximal margin without bulbous protrusion, merus 1.3 times as long as wide, 0.6 as long as ischium; carpus 1.4 times as long as wide, 0.5 as long as ischium, without bulbous protrusion; propodus 2.1 times as long as wide, 0.7 as long as ischium; dactylus slender, 1 as long as propodus, 2.9 times as long as basal width.

Pleopods without setae, exopod larger than endopod.

Uropod more than half the length of pleotelson, peduncle lateral margin without setae; rami not extending beyond pleotelson, marginal setae absent, apices narrowly rounded. Endopod apically rounded, 3.7 times as long as greatest width, lateral margin weakly convex, medial margin weakly convex, terminating with 0 setae. Exopod extending beyond end of endopod, 6.0 times as long as greatest width, apically rounded, lateral margin weakly convex, medial margin weakly convex, terminating without setae.

**Fig. 18:**
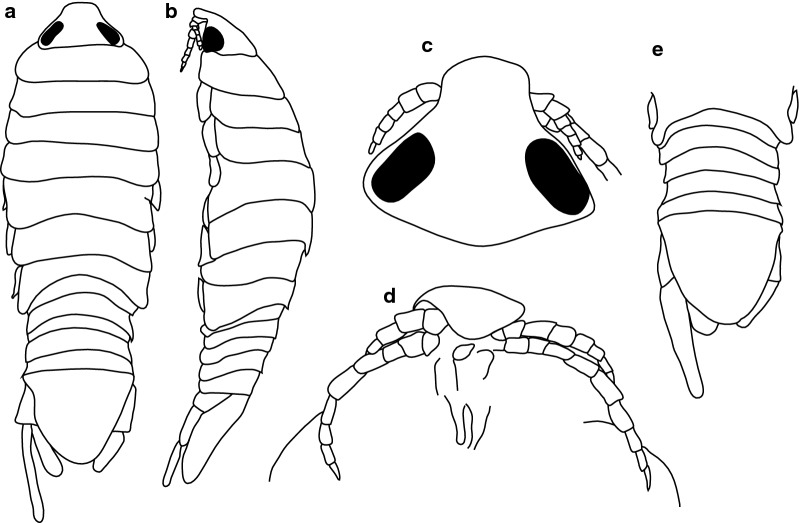
*Anilocra angeldaviesae* n. sp. ♂ (30.0 × 9.0, paratype, BMNH1924.5.30.7). **a** Dorsal view. **b** Lateral view. **c** Dorsal view of cephalon. **d** Ventral view of cephalon. **e** Pleotelson

***Male***. [BMNH1924.5.30.7–10; Fig. 18.] Size 30 × 18. Similar to female but much smaller. Body ovoid to rectangular, weakly twisted, 3.2 times as long as wide. Pleopod 2 *appendix masculina* with parallel margins, distally narrowly rounded.

**Remarks**

*Anilocra angeladaviesae* n. sp. can best be identified by the distinct elongate lateral margin on the third article of the antenna peduncle that appears to partially cover the lateral margin of the fourth article; the antennule is nearly half the length of the antenna; the body is ovoid and pereonites 1–3 narrow weakly towards the cephalon.

It is likely that the specimens examined here are those mentioned by Monod as *A. capensis* from Morocco [35] because the material examined from NHM was collected by Monod in 1923, and is clearly labelled as *A. capensis* from Morocco. Monod [35] referenced the genus names, *Sama* and *Dentex*, and the species, *Morone punctata* (current accepted name is *Dicentrarchus punctatus* Bloch, 1792).

Compared to Schioedte & Meinert’s [7] drawings of *A. physodes*, the pereonite margins and pleotelson of *A. angeladaviesae* n. sp. are much straighter and less acute, respectively. Compared to *A. capensis*, *A. angeladaviesae* n. sp. has a narrower pleonite 1 in some specimens. *Anilocra angeladaviesae* n. sp. lacks the curved lateral margin of the pereopod 6 propodus, which is present on both *A. capensis* and *A. bunkleywilliamsae* n. sp. *Anilocra bunkleywilliamsae* n. sp. also has large dense chromatophores that are not present on *A. angeladaviesae* n. sp. *Anilocra paulsikkeli* n. sp. and *A. jovanasi* n. sp. are both more elongate and have nodules on pereopods 1 and 2 compared to *A. angeladaviesae* n. sp. *Anilocra angeladaviesae* n. sp. has a more rounded rostrum and posterior margin of pereonite 7 and more ovoid body compared to *A. hadfieldae* n. sp.

***Anilocra hadfieldae*** **n. sp.**

***Type-host***: Unknown.

***Type-locality***: Cape Blanco, Gambia.

***Type-material***: Holotype ♀ (42.0 × 16.0, BMNH1952.9.9.29–30); paratype ♂ (30.0 × 9.0, BMNH1952.9.9.29–30). Collected by M. Routh, 1952.

***ZooBank registration***: The Life Science Identifier (LSID) for *Anilocra hadfieldae* n. sp. is urn:lsid:zoobank.org:act:9744DBAC-6A5C-4C5A-A622-D96F0A1C9F0A.

***Etymology***: This species is named for Dr Kerry Ann Hadfield in gratitude for her expertise in cymothoid taxonomy and for making RL Welicky’s postdoctoral tenure in South Africa a truly memorable and valuable experience to her both academically and personally.

**Description**

**Fig. 19:**
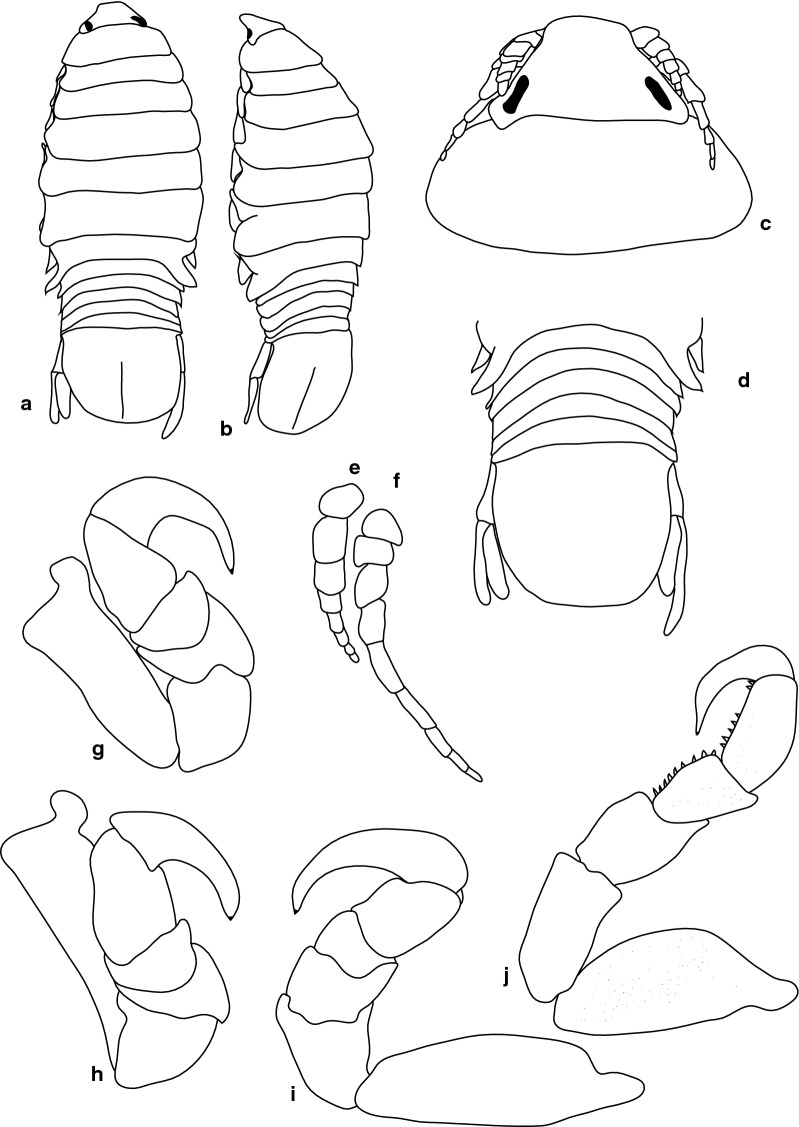
*Anilocra hadfieldae* n. sp. ♀ (42.0 × 16.0, holotype, BMNH1952.9.9.29-30). **a** Dorsal view. **b** Lateral view. **c** Dorsal view of cephalon. **d** Pleotelson. **e** Antennula. **f** Antenna. **g** Pereopod 1. **h** Pereopod 2. **i** Pereopod 6. **j** Pereopod 7

***Female***. [BMNH1952.9.9.29–30; Fig. 19.] Size 42.0 × 16.0. Body ovoid, weakly twisted, 2.6 times as long as greatest width, dorsal surfaces smooth and polished in appearance, widest at pereonite 5, most narrow at pereonite 1, pereonite lateral margins mostly posteriorly ovate. Cephalon 0.52 as long as wide as measured in dorsal view, trapezoid-shaped. Eyes oval, with distinct margins, one eye width 0.1 times width of cephalon; one eye length 0.4 times length of cephalon. Pereonite 1 in line with base of cephalon, smooth, anterior border straight, anterolateral angle narrowly rounded. Posterolateral margins of pereonites smooth and straight, posterolateral margin of pereonite 7 acute and extending in an arc abruptly laterally and dorsally. Coxae 2–3 similar width to coxae 4–7; with posteroventral angles rounded; coxae 4–7 with rounded point and curved; not extending past posterior pereonite margin. Pereonites 1–5 increasing in length and width; pereonites 6–7 decreasing in length and width; pereonites 5 and 6 subequal, pereonites 1–4 subequal. Pleon with pleonite 1 wider than pleonites 2–4, visible in dorsal view; posterior margin of pleonites 1–4 posteriorly concave, pleonite 5 posteriorly slightly concave to straight, mostly concave. Pleonite 2 not overlapped by pereonite 7; posterolateral angles of pleonite 2 narrowly rounded. Pleonite 1 similar in form to pleonites 2 and 3. Pleonites 3–5 similar in form to pleonite 2; pleonite 5 not extended posteriorly and equal width to pleonite 4, posterior margin straight. Pleotelson 0.9 times as long as anterior width, dorsal surface with lateral indent, lateral margins weakly convex, posterior margin rounded, with medial indent.

Antennula approximately as stout as antenna, comprised of 8 articles; peduncle articles 1 and 2 distinct and articulated; article 2 1.3 times as long as article 1; article 3 1 times as long as wide, 0.4 times as long as combined lengths of articles 1 and 2; flagellum with 5 articles, extending to middle of eye. Antenna comprised of 9 articles; peduncle article 3 1.6 times as long as article 2; article 4 1.4 times as long as wide, 0.9 times as long as article 3; article 5 2.8 times as long as wide, 1.4 times as long as article 4; flagellum with 4 articles, extending to middle of pereonite 1.

Pereopod 1 basis 2.4 times as long as greatest width; ischium 0.42 times as long as basis; merus proximal margin without bulbous protrusion; carpus with straight proximal margin; propodus 1.5 times as long as wide; dactylus stout, 1.4 as long as propodus, 2.4 times as long as basal width. Pereopod 2 propodus 1.7 as long as wide; dactylus 1.2 as long as propodus. Pereopods gradually increasing in size towards posterior. Pereopod 6 basis 3.2 times as long as greatest width, ischium 0.5 times as long as basis, propodus 1.4 as long as wide, dactylus 1.6 as long as propodus. Pereopod 7 basis 2.8 times as long as greatest width; ischium 0.6 as long as basis, without protrusions; 0.8 as long as ischium; carpus 1.6 times as long as wide, 0.7 as long as ischium, without bulbous protrusion; propodus 1.9 times as long as wide, 0.8 as long as ischium; dactylus stout, 0.9 as long as propodus, 2.9 times as long as basal width.

Pleopods without setae, exopod larger than endopod.

Uropod peduncle 0.5 times as long as rami, lateral margin without setae; rami not extending beyond pleotelson, marginal setae absent, apices narrowly rounded. Endopod apically rounded, 5 times as long as greatest width, lateral margin weakly convex, medial margin weakly convex, terminating with 0 setae. Exopod extending to end of endopod or extending beyond end of endopod.

**Fig. 20:**
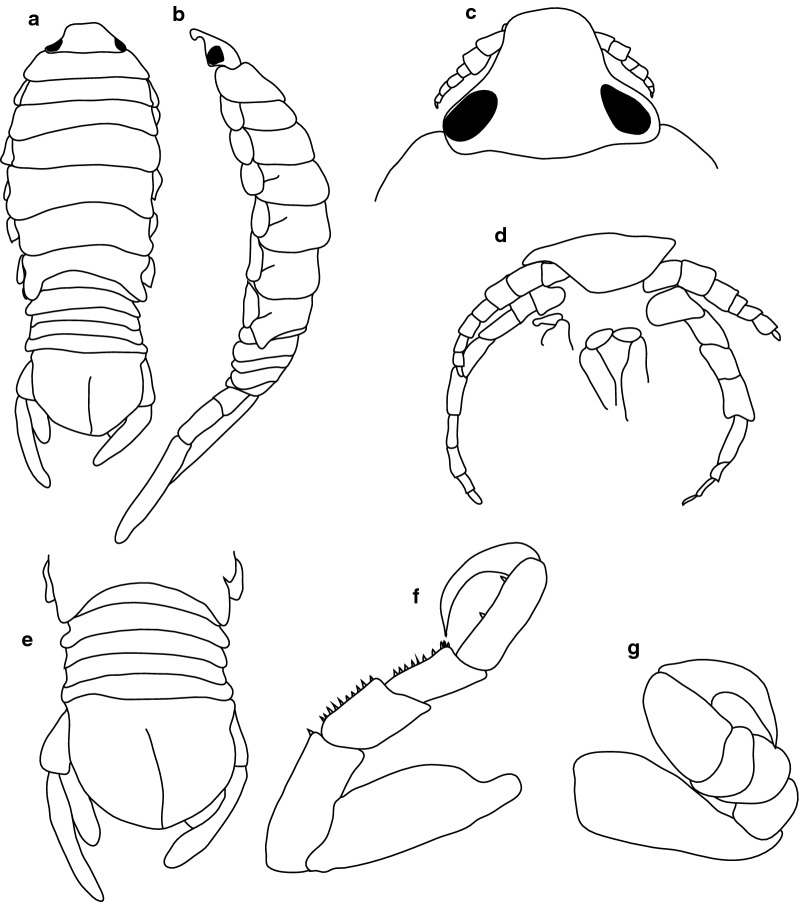
*Anilocra hadfieldae* n. sp. ♂ (30.0 × 9.0, paratype, BMNH1952.9.9.29-3). **a** Dorsal view. **b** Lateral view. **c** Dorsal view of cephalon. **d** Ventral view of cephalon. **e** Pleotelson. **f** Pereopod 7. **g** Pereopod 1

***Male***. [BMNH1952.9.9.29–30; Fig. 20.] Size 30.0 × 9.0. Male similar to female but smaller. Body weakly twisted, ovoid, 2.9 times as long as wide. Pleopod 2 *appendix masculina* with parallel margins, distally narrowed.

**Remarks**

*Anilocra hadfieldae* n. sp. has a distinct body shape. The body is more parallel than ovoid and the posterior lateral margins of pereonite 7 are substantially more acute than round and more obviously bent upward; pereopods 7 have small and light pigmentation and the pleotelson has a distinct medial indentation. The eyes are proportionally narrower than the other species described above.

The antenna and antennula of *Anilocra hadfieldae* n. sp. differ from that of *A. capensis,* the latter being stouter. The antenna peduncle articles of *A. hadfieldae* n. sp. have more parallel lateral margins than those of *A. angeladaviesae* n. sp. *Anilocra hadfieldae* n. sp. has no nodules or curved lateral produced margins of the pereopods, making it distinct from *A. capensis*, *A. bunkleywilliamsae* n. sp., *A. paulsikkeli* n. sp., and *A. jovanasi* n. sp.

**Key to the African species of** ***Anilocra***

1a Body weakly twisted ……………………………………………………………………………………………………. 2

1b Body straight ……………………………………………………………………………………………………………… 3

2a Posterolateral margins of pleonite 5 extended posteriorly, overlaying lateral margins of pleotelson …………………………………………………………………………………………….. *A. jovanasi* n. sp.

2b Posterolateral margins of pleonite 5 not extended posteriorly, not overlaying lateral margins of pleotelson, posterolateral margins of pereonite 7 acute, extending in an arc abruptly laterally and dorsally …………………………………………………………………………………………… *A. hadfieldae* n. sp.

3a Body elongate, pleonite 1 concealed by pereonite 7 …………………………………………………………. 4

3b Body ovoid, pleonite 1 not concealed by pereonite 7 ……………………………………………………….. 5

4a Pleonite 1 substantially concealed by pereonite 7; body 3.7 times as long as wide; pleotelson lateral margins subparallel *A. paulsikkeli* n. sp.

4b Pleonite 1 partly concealed by pereonite 7, body 3.5 times as long as wide, pleotelson lateral margins convex *A. leptosoma* Bleeker, 1857

5a Pereonites 1–3 strongly narrowed ………………………………………………………… *A. ianhudsoni* n. sp.

5b Pereonites 1–3 weakly narrowed ……………………………………………………………………………………. 6

6a Lateral margin on article 3 of antenna peduncle elongate, extending onto lateral margin of article 4; pereopod 6 propodus not inflated *A. angeladaviesae* n. sp.

6b Lateral margin on article 3 of antenna peduncle not elongate, not extending onto lateral margin of article 4; pereopod 6 propodus with produced curvature 7

7a Body 2.2 times as long as greatest width; article 3 of antennula peduncle 1.2 times as long as wide; dense chromatophores present on pereopods *A. bunkleywilliamsae* n. sp.

7b Body 2.4–2.6 times as long as greatest width; article 3 of antennula peduncle article 0.8 times as long as wide; dense chromatophores absent from pereopods *A. capensis* Leach, 1818

## Discussion

*Anilocra capensis* has been perhaps the most utilised (but not necessarily correct) name for any species of *Anilocra* in South Africa and likely in other areas of Africa, too. This is not surprising as only two drawings of adult female *A. capensis* existed before the present work. With few data on *A. capensis* and other African species, it was a logical pitfall to identify species of *Anilocra* collected from South Africa and elsewhere in Africa as *A. capensis*. This paper provides a detailed description of one of the original named species, and descriptions of six species new to science and thus will aid in the correct identification of these organisms. It is important to note that our molecular findings were unexpected as the nucleotide- and p-distance values between *A. capensis* and other *Anilocra* showed at levels more similar to differences observed between genera [24]. In order to better quantify species and genus level differences within the family Cymothoidae, multi-gene phylogenetic analyses of old and new world species that are specifically linked to correct morphological identification are needed.

## Conclusions

The findings of this work should greatly reduce further misidentification of *A. capensis.* We provide morphological drawings and quantitative and qualitative descriptions of this species, as well as relevant comparisons among the six new species of *Anilocra* from Africa. By providing the first molecular data for *A. capensis*, we also contribute to improving the identification of cryptic species *via* molecular analyses. We also demonstrate the value of morphological data as we found an ample number of morphological differences among the closely similar species described herein. In particular, our *A. capensis* redescription validates the name *A. capensis*, and provides critical baseline data to understand species differences of African species of *Anilocra*.

## Data Availability

Data supporting the conclusions of this article are included within the article. The sequences were submitted to the GenBank database under the Accession Numbers MK450443-MK450452. The type-material per species is deposited in the museum from which it was collected.

## Abbreviations

NHM: The Natural History Museum of London, UK
SAM, SAMC: The iZiko South Africa Museum, Cape Town, South Africa
BLASTn: Basic Local Alignment Search Tool for nucleotides
*cox*1: mitochondrial cytochrome *c* oxidase subunit 1 gene
